# A Phase 3 Open-label, Randomized, Controlled Study to Evaluate the Efficacy and Safety of Intravenously Administered Ravulizumab Compared with Best Supportive Care in Patients with COVID-19 Severe Pneumonia, Acute Lung Injury, or Acute Respiratory Distress Syndrome: A structured summary of a study protocol for a randomised controlled trial

**DOI:** 10.1186/s13063-020-04548-z

**Published:** 2020-07-13

**Authors:** Keisha Smith, Amy Pace, Stephan Ortiz, Shamsah Kazani, Scott Rottinghaus

**Affiliations:** 1 Medical Writing, Alexion Pharmaceuticals, Boston, MA USA; 2 Quantitative Sciences, Alexion Pharmaceuticals, Boston, MA USA; 3 Clinical Pharmacology, Alexion Pharmaceuticals, Boston, MA USA; 4 Department of Clinical Development Sciences, Alexion Pharmaceuticals, Inc., 121 Seaport Boulevard, Boston, MA 02210 USA

**Keywords:** COVID-19, severe acute respiratory distress syndrome coronavirus 2, lung injury, acute respiratory distress syndrome, respiratory distress syndrome, severe pneumonia, severe acute respiratory syndrome, acute pneumonia, viral, ravulizumab, antibodies, monoclonal, humanized, complement, complement inhibitor, Ultomiris, hospitalization, randomized controlled study, adult

## Abstract

**Objectives:**

Primary Objective

• To evaluate the effect of ravulizumab, a long-acting complement (C5) inhibitor plus best supportive care (BSC) compared with BSC alone on the survival of patients with COVID-19.

Secondary Objectives

• Number of days free of mechanical ventilation at Day 29

• Duration of intensive care unit stay at Day 29

• Change from baseline in Sequential Organ Failure Assessment (SOFA) score at Day 29

• Change from baseline in peripheral capillary oxygen saturation/ fraction of inspired oxygen (SpO2 /FiO2) at Day 29

• Duration of hospitalization at Day 29

• Survival (based on all-cause mortality) at Day 60 and Day 90

Safety

• Incidence of treatment-emergent adverse events and treatment-emergent serious adverse events.

PK/PD/Immunogenicity

• Change in serum ravulizumab concentrations over time

• Change in serum free and total C5 concentrations over time

• Incidence and titer of anti-ALXN1210 antibodies

Biomarkers

• Change in absolute level of soluble biomarkers in blood associated with complement activation, inflammatory processes, and hypercoagulable states over time

Exploratory

• Incidence of progression to renal failure requiring dialysis at Day 29

• Time to clinical improvement (based on a modified 6-point ordinal scale) over 29 days

• SF-12 Physical Component Summary (PCS) and Mental Component Summary (MCS) scores at Day 29 (or discharge), Day 60, and Day 90

• EuroQol 5-dimension 5-level (EQ-5D-5L) scores at Day 29 (or discharge), Day 60, and Day 90

**Trial design:**

This is a multicenter Phase 3, open-label, randomized, controlled, study.

The study is being conducted in acute care hospital settings in the United States, United Kingdom, Spain, France, Germany, and Japan.

**Participants:**

Male or female patients at least 18 years of age, weighing ≥ 40 kg, admitted to a designated hospital facility for treatment will be screened for eligibility in this study.

Key Inclusion criteria

• Confirmed diagnosis of SARS-CoV-2 infection (eg, via polymerase chain reaction [PCR] and/or antibody test) presenting as severe COVID-19 requiring hospitalization

• Severe pneumonia, acute lung injury, or ARDS confirmed by computed tomography (CT) or X-ray at Screening or within the 3 days prior to Screening, as part of the patient’s routine clinical care

• Respiratory distress requiring mechanical ventilation, which can be either invasive (requiring endotracheal intubation) or non-invasive (with continuous positive airway pressure [CPAP] or bilevel positive airway pressure [BiPAP])

Key Exclusion criteria

• Patient is not expected to survive for more than 24 hours

• Patient is on invasive mechanical ventilation with intubation for more than 48 hours prior to Screening

• Severe pre-existing cardiac disease (ie, NYHA Class 3 or Class 4, acute coronary syndrome, or persistent ventricular tachyarrhythmias)

• Patient has an unresolved *Neisseria meningitidis* infection

Excluded medications and therapies

• Current treatment with a complement inhibitor

• Intravenous immunoglobulin (IVIg) within 4 weeks prior to randomization on Day 1

Excluded prior/concurrent clinical study experience

• Treatment with investigational therapy in a clinical study within 30 days before randomization, or within 5 half-lives of that investigational therapy, whichever is greater

• Exceptions

a. Investigational therapies will be allowed if received as part of best supportive care through an expanded access protocol or emergency approval for the treatment of COVID-19.

b. Investigational antiviral therapies (such as remdesivir) will be allowed even if received as part of a clinical study.

**Intervention and comparator:**

The study consists of a Screening Period of up to 3 days, a Primary Evaluation Period of 4 weeks, a final assessment at Day 29, and a Follow-up Period of 8 weeks. For patients randomized to ravulizumab plus BSC, a weight-based dose of ravulizumab (≥40 to < 60 kg/2400 mg, 60 to < 100 kg/2700 mg, ≥ 100 kg/3000 mg) will be administered on Day 1. On Day 5 and Day 10, additional doses of 600 mg (≥40 to <60 kg) or 900 mg (>60 kg) ravulizumab will be administered and on Day 15 patients will receive 900 mg ravulizumab. There is no active or placebo comparator in this open-label clinical trial. The total duration of each patient’s participation is anticipated to be approximately 3 months.

**Main outcomes:**

The primary efficacy outcome of this study is survival (based on all-cause mortality) at Day 29.

**Randomisation:**

Patients will be randomized in a 2:1 ratio (ravulizumab plus BSC:BSC alone). Randomization will be stratified by intubated or not intubated on Day 1. Computer-generated randomization lists will be prepared by a third party under the direction of the sponsor. Investigators, or designees, will enrol patients and then obtain randomization codes using an interactive voice/web response system. The block size will be kept concealed so that investigators cannot select patients for a particular treatment assignment.

**Blinding (masking):** This is an open-label study.

**Numbers to be randomised (sample size):** Approximately 270 patients will be randomly assigned in a 2:1 ratio to ravulizumab plus BSC (n=180) or BSC alone (n=90).

**Trial status:**

Protocol Number: ALXN1210-COV-305

Original Protocol: 09 Apr 2020

Protocol Amendment 1 (Global): 13 Apr 2020

Protocol Amendment 2 (Global): 17 Apr 2020

Protocol Amendment 3 (Global): 09 Jun 2020

Recruitment is currently ongoing.

Recruitment was initiated on 11 May 2020.

We expect recruitment to be completed by 30 Nov 2020.

**Trial registration:**

Clinicaltrials.gov: Protocol Registry Number: NCT04369469; First posted; 30 Apr 2020

EU Clinical Trials Register: EudraCT Number: https://www.clinicaltrialsregister.eu/ctr-search/search?query=ALXN1210-COV-305, Start date: 07 May 2020

**Full protocol:**

The full redacted protocol is attached as an additional file, accessible from the Trials website (Additional file 1). In the interest in expediting dissemination of this material, the familiar formatting has been eliminated; this Letter serves as a summary of the key elements of the full protocol.

## Supplementary information

**Additional file 1.** Full Study Protocol.

## Data Availability

Upon publication of the primary manuscript, Alexion will consider requests for disclosure of clinical study participant-level data provided that participant privacy is assured through methods like data de-identification, pseudonymization, or anonymization (as required by applicable law), and if such disclosure was included in the relevant study informed consent form or similar documentation. Qualified academic investigators may request participant-level clinical data and supporting documents (statistical analysis plan and protocol) pertaining to Alexion-sponsored studies. Further details regarding data availability and instructions for requesting information are available in the Alexion Clinical Trials Disclosure and Transparency Policy at http://alexion.com/research-development. Link to Data Request Form (https://alexion.com/contact-alexion/medical-information)

