# Supplementary material for: A Phase 3 Open-label, Randomized, Controlled Study to Evaluate the Efficacy and Safety of Intravenously Administered Ravulizumab Compared with Best Supportive Care in Patients with COVID-19 Severe Pneumonia, Acute Lung Injury, or Acute Respiratory Distress Syndrome: A structured summary of a study protocol for a randomised controlled trial
Source: Trials. 2020 Jul 13;21:639. doi: 10.1186/s13063-020-04548-z (PMC7355517; doi:10.1186/s13063-020-04548-z)
Supplement: Supplementary file 1 — Additional file 1. Full Study Protocol. [file 13063_2020_4548_MOESM1_ESM.pdf]

TITLE PAGE

Protocol Title: A Phase 3 Open-label, Randomized, Controlled Study to Evaluate the Efficacy and Safety of Intravenously Administered Ravulizumab Compared with Best Supportive Care in Patients with COVID-19 Severe Pneumonia, Acute Lung Injury, or Acute Respiratory Distress Syndrome

Protocol Number: ALXN1210-COV-305

Compound: Ravulizumab (ALXN1210)

Study Phase: Phase 3

Short Title: Efficacy and Safety Study of IV Ravulizumab in Patients with COVID-19 Severe Pneumonia

Sponsor Name: Alexion Pharmaceuticals, Inc.

Legal Registered Address:

Alexion Pharmaceuticals, Inc.

121 Seaport Boulevard

Boston, MA 02210

USA

Regulatory Agency Identifier Number(s)

IND: 149,271

EudraCT: 2020-001497-30

Original Protocol: 09 Apr 2020

Protocol Amendment 1 (Global): 13 Apr 2020

Protocol Amendment 2 (Global): 17 Apr 2020

Protocol Amendment 2.1 (Germany): 08 May 2020

Protocol Amendment 2.2 (France): 08 May 2020

Protocol Amendment 2.3 (France): 13 May 2020

Protocol Amendment 3 (Global): 09 Jun 2020

SPONSOR SIGNATORY

[redacted]

June 10, 2020

Date

Medical Monitor Name and Contact Information:

[redacted]
121 Seaport Boulevard
Boston MA 02210 USA
direct [redacted]
mobile

INVESTIGATOR'S AGREEMENT

I have read the study protocol and agree to conduct the study in accordance with this protocol, all applicable government regulations, the principles of the International Council for Harmonisation of Technical Requirements for Pharmaceuticals for Human Use (ICH) E6 Guidelines for Good Clinical Practice, and the principles of the World Medical Association Declaration of Helsinki. I also agree to maintain the confidentiality of all information received or developed in connection with this protocol.

____

Printed Name of Investigator

____

Signature of Investigator

____

Date

TABLE OF CONTENTS

TITLE PAGE 1
SPONSOR SIGNATORY 2
INVESTIGATOR'S AGREEMENT 3
TABLE OF CONTENTS 4
LIST OF TABLES 9
LIST OF FIGURES 10
PROTOCOL AMENDMENT SUMMARY OF CHANGES TABLE 11
1. PROTOCOL SUMMARY 18
1.1. Synopsis 18
1.2. Schema 21
1.3. Schedule of Activities 22
2. INTRODUCTION 26
2.1. Study Rationale 26
2.2. Background 26
2.2.1. Nonclinical Evidence Supporting the Role of Complement in COVID-19 26
2.2.2. Clinical Hypothesis Supporting Utility of Ravulizumab in COVID-19 28
2.3. Benefit/Risk Assessment 29
2.3.1. Risk Assessment 29
2.3.2. Benefit Assessment 30
2.3.3. Overall Benefit: Risk Conclusion 30
3. OBJECTIVES AND ENDPOINTS 32
4. STUDY DESIGN 33
4.1. Overall Design 33
4.2. Scientific Rationale for Study Design 33
4.3. Justification for Dose 35
4.4. End of Primary Evaluation Definition 38
4.5. End of Study Definition 38
5. STUDY POPULATION 39
5.1. Inclusion Criteria 39
5.2. Exclusion Criteria 39
5.3. Lifestyle Considerations 40

5.4. Screen Failures 40
6. STUDY INTERVENTION 41
6.1. Study Intervention(s) Administered 41
6.1.1. Packaging and Labeling 41
6.2. Preparation/Handling/Storage/Accountability 42
6.3. Measures to Minimize Bias: Randomization and Blinding 43
6.4. Study Intervention Compliance 43
6.5. Concomitant Therapy 43
6.5.1. Disallowed Medicine and Therapy 43
6.6. Dose Modification 43
6.7. Intervention After the End of the Study 43
7. DISCONTINUATION OF STUDY INTERVENTION AND PATIENT DISCONTINUATION/WITHDRAWAL 44
7.1. Discontinuation of Study Intervention 44
7.2. Patient Discontinuation/Withdrawal from the Study 44
7.3. Lost to Follow-up 44
8. STUDY ASSESSMENTS AND PROCEDURES 46
8.1. Eligibility and Administrative Assessments 46
8.1.1. Informed Consent 46
8.1.2. Inclusion and Exclusion Criteria 46
8.1.3. Medical History 46
8.1.4. Demographics 46
8.1.5. Vaccination or Antibiotic Prophylaxis 47
8.2. Screening Assessments 47
8.2.1. SARS-COV-2 Testing 47
8.2.2. Chest Computed Tomography or X-ray 47
8.2.3. Pregnancy Testing 47
8.3. Efficacy Assessments 47
8.3.1. Primary Efficacy Assessments 47
8.3.2. Secondary Efficacy Assessments 47
8.3.2.1. Sequential Organ Failure Assessment Score 48
8.4. Safety Assessments 48
8.4.1. Physical Examinations 48

8.4.2. Vital Sign Measurements 49
8.4.3. Electrocardiograms 49
8.4.4. Clinical Safety Laboratory Assessments 49
8.4.5. Immunogenicity 50
8.4.6. Glasgow Coma Scale 50
8.4.7. Pregnancy 50
8.4.8. Patient Safety Information Card 51
8.4.9. Vaccine and Antibiotic Prophylaxis 51
8.4.10. Hospital Discharge 51
8.4.11. Follow-up Monitoring 52
8.4.12. Early Termination 52
8.5. Exploratory Assessments 52
8.5.1. Clinical Improvement at Day 29 52
8.5.2. 12-item Short Form at Days 29, 60, and 90 52
8.5.3. EuroQol-5 Dimension-5 Level at Days 29, 60, and 90 53
8.6. Adverse Events and Serious Adverse Events 53
8.6.1. Time Period and Frequency for Collecting AE and SAE Information 54
8.6.2. Method of Detecting AEs and SAEs 54
8.6.3. Follow-up of AEs and SAEs 54
8.6.4. Regulatory Reporting Requirements for SAEs 54
8.6.5. Adverse Events of Special Interest 55
8.7. Treatment of Overdose 55
8.8. Pharmacokinetics 55
8.9. Pharmacodynamics 55
8.10. Genetics 55
8.11. Biomarkers 56
8.12. Immunogenicity 56
8.13. Health Economics Data or Medical Resource Utilization 56
9. STATISTICAL CONSIDERATIONS 57
9.1. Statistical Hypotheses 57
9.2. Sample Size Determination 57
9.3. Populations for Analyses 58
9.4. Statistical Analyses 58

9.4.1. Efficacy Analyses 59
9.4.1.1. Analyses of Primary Efficacy Endpoint 59
9.4.1.2. Analyses of Secondary Efficacy Endpoints 59
9.4.1.3. Multiplicity Adjustment 60
9.4.2. Safety Analyses 60
9.4.3. Pharmacokinetic and Pharmacodynamic Analyses 61
9.4.4. Biomarker Analyses 61
9.4.5. Immunogenicity Analyses 61
9.4.6. Analyses of Exploratory Endpoints 61
9.5. Interim Analyses 61
9.5.1. Data Monitoring Committee 62
10. SUPPORTING DOCUMENTATION AND OPERATIONAL CONSIDERATIONS 63
10.1. Appendix 1: Regulatory, Ethical, and Study Oversight Considerations 63
10.1.1. Regulatory and Ethical Considerations 63
10.1.2. Financial Disclosure 63
10.1.3. Informed Consent Process 64
10.1.4. Data Protection 65
10.1.5. Dissemination of Clinical Study Data 65
10.1.6. Data Quality Assurance 65
10.1.7. Source Documents 66
10.1.8. Study and Site Start and Closure 66
10.1.9. Publication Policy 67
10.2. Appendix 2: Clinical Laboratory Tests 68
10.3. Appendix 3: Adverse Events: Definitions and Procedures for Recording, Evaluating, Follow-up, and Reporting 70
10.3.1. Definition of AE 70
10.3.2. Definition of SAE 71
10.3.3. Recording and Follow-Up of AE and/or SAE 71
10.3.4. Reporting of SAEs 72
10.4. Appendix 4: Contraceptive Guidance and Collection of Pregnancy Information 74
10.4.1. Definitions 74

10.4.2. Contraception Guidance 74
10.4.2.1. Guidance for Female Patients 74
10.4.2.2. Guidance for Male Patients 76
10.4.3. Collection of Pregnancy Information 76
10.4.3.1. Male Patients With Partners Who Become Pregnant 77
10.4.3.2. Female Patients Who Become Pregnant 77
10.5. Appendix 5: Biomarkers 78
10.6. Appendix 6: Management of Potential Drug Infusion Reactions 79
10.7. Appendix 7: Protocol Amendment History 81
10.8. Appendix 8: Abbreviations 82
11. REFERENCES 84

LIST OF TABLES

Table 1: Schedule of Activities 22
Table 2: Potential Risks and Mitigation Strategies 29
Table 3: Objectives and Endpoints 32
Table 4: Study Design Rationale for Study ALXN1210-COV-305 33
Table 5: Ravulizumab Dosage Regimen for COVID-19 Severe Pneumonia, Acute Lung Injury, or Acute Respiratory Distress Syndrome 41
Table 6: Ravulizumab Dosage Form and Strength 42
Table 7: Sequential Organ Failure Assessment Scoring 48
Table 8: Glasgow Coma Scale 50
Table 9: Protocol-required Laboratory Assessments 68
Table 10: Clinical Criteria for Diagnosing Anaphylaxis 80

LIST OF FIGURES

Figure 1: Study ALXN1210-COV-305 Schematic 21

Figure 2: Eculizumab Clearance Changes Over Time in Pediatric Patients With Thrombotic Microangiopathy Following Hematopoietic Stem-cell Transplant 35

Figure 3: Simulations Comparing "With" and "Without" the Faster Clearance Assumption of Ravulizumab in Adult Patients With Thrombotic Microangiopathy Following Hematopoietic Stem-cell Transplant 37

PROTOCOL AMENDMENT SUMMARY OF CHANGES TABLE

Amendment 3 (Global)

Overall Rationale for the Amendment:

This global amendment was initiated to update the inclusion and exclusion criteria, study endpoints and objectives, and the schedule of activities. Patient-reported outcomes (SF-12 and EQ-5D-5L) were added and changes were also implemented to align content in Section 9 (Statistical Considerations) with version 1and version 2 of the statistical analysis plan.

This amendment is considered to be substantial based on the criteria set forth in Article 10(a) of Directive 2001/20/EC of the European Parliament and the Council of the European Union. Substantive changes are documented in the table below.

Section # and Title	Description of Change	Brief Rationale
1.1 – Synopsis,<br>3 – Objectives and Endpoints<br>8.3.2 – Secondary Efficacy Assessments	• Reordered secondary endpoints	The ordering of testing for the secondary endpoints has been changed to align with the order of interest from a clinical and patient perspective. This reordering reduces the risk of a type II error if a more clinically important endpoint is set below another endpoint in the hierarchy for which the null hypothesis is not rejected.<br><br>The secondary endpoint of survival at Day 60 and Day 90 has been removed from the testing procedure when all patients have completed the Primary Evaluation Period, which is Day 29, as sufficient information for this endpoint may not be available.
1.1 – Synopsis,<br>3 – Objectives and Endpoints<br>8.3.2 – Secondary Efficacy Assessments<br>9.4 – Statistical Analyses	• Revised biomarker endpoint<br><br>• Added 3 exploratory objectives to evaluate: the effect of ravulizumab + BSC or BSC alone on clinical improvement, and health-related quality of life (SF-12 PCS and MCS, and EQ-5D-5L scores)<br><br>• Redefined baseline	• To include an evaluation of prothrombic activity<br><br>• Clinical improvement as measured via a 6-point ordinal scale is included to evaluate the severity of the underlying disease over time; the SF-12 and EQ-5D-5L are patient-reported outcome questionnaires that are included to assess health-related quality of life<br><br>• To align with revised definition in version 1 of the SAP
1.1 – Synopsis (Intervention Groups and Duration)<br>6.1 – Study Interventions Administered	Added dosing units to the header columns of Table s1 and Table 5	Correction of a typographical error
1.2 – Schema	• Revised schema to add all study timepoints for both treatment groups<br>• Revised footnotes	For clarity and parallel construction
1.3 – Schedule of Activities<br>8.13 – Health Economics Data or Medical Resource Utilization	Added SF-12 and EQ-5D-5L questionnaires	For internal document consistency as questionnaires were added as exploratory endpoints
1.3 – Schedule of Activities	Added timepoints at Screening for “Mechanical ventilation status” and “Adverse event review and evaluation”	To ensure AEs are collected after signing of the informed consent form<br><br>To ensure that mechanical ventilation status is assessed during screening
1.3 – Schedule of Activities	Added timepoint at Day 60 and Day 90 for assessment of concomitant medications and nonpharmacologic treatments and therapies	For clarity

1.3 – Schedule of Activities	Footnote #4: Added sentence that patients who are discontinued from the study will be contacted by telephone on Day 29 to assess their health status	To determine health status (including survival) at Day 29
1.3 – Schedule of Activities	· Updated SoA to indicate that FiO2, SpO2, PaO2 will be assessed daily<br><br>· Footnote #9: Added sentence that SpO2, PaO2 (if available), and FiO2 should be measured predose for patients randomized to ravulizumab + BSC	· For clarity<br><br>· To establish baseline values for these parameters
1.3 – Schedule of Activities	Footnote #14: Revised to ensure that immunogenicity sampling is not collected postdose	For clarity
	Footnote #14 and #15: Expanded the predose (within 4 hours of the start of infusion) and postdose (within 4 hours after the end of infusion) sampling windows	Given the half-life of the molecule, these expanded windows allow the investigational site staff to collect protocol-specified laboratory samples within a reasonable timeframe.
1.3 – Schedule of Activities	Footnote #16: Revised to indicate that biomarker samples will be collected for all patients; however, for patients randomized to the ravulizumab + BSC treatment group biomarker samples will be collected any time before the start of infusion	For clarity
1.3 – Schedule of Activities	Footnote #19: Added statement to indicate that medical history should include the date of first onset of signs and symptoms of SARS-CoV-2 infection.	For clarity
5 – Study Population	Added a clarifying statement that the 3-day Screening Period is allowed to evaluate patients for eligibility. If the Screening and Day 1 Visits are combined, the patient has to meet all inclusion/no exclusion criteria prior to randomization.	To clarify that patients may be evaluated for eligibility at any time during the 3-day Screening Period.
5.1 – Inclusion Criteria<br>8.2.1 - SARS-COV-2 Testing	Revised criterion #2 – Confirmed diagnosis of SARS-CoV-2 infection can be via PCR and/or antibody test	For clarity
5.1 – Inclusion Criteria	Revised criterion #4 – Target population is required to be in respiratory distress with either invasive or noninvasive mechanical ventilation (also defined)	For clarity and to ensure investigational sites are supplied with a clear definition of invasive and noninvasive mechanical ventilation

5.2 – Exclusion Criteria,<br>6.5.1 – Disallowed Medicine and Therapy	Removed rituximab and mitoxantrone as prohibited medications (criteria 5b and 5c)	These medications have been historically disallowed in the Sponsor's neurology studies due to a potential drug-drug interaction that could lower rituximab levels, and due to a potential confounding effect for efficacy with mitoxantrone. However, there is a very low likelihood that patients with severe COVID-19 will be receiving these therapies in an acute setting, and the potential interactions are less important in this acute setting. Therefore, the Sponsor has elected to remove these medications from the list of excluded concomitant medications.
5.2 – Exclusion Criteria<br>6.5.1 – Disallowed Medicine and Therapy	Revised washout period for IVIg (criterion 5d)	For clarity
5.2 – Exclusion Criteria	Revised criterion to indicate that 1) patients who receive medications as part of BSC at the hospital due to emergency authorization under a compassionate use or expanded access program and 2) patients who receive antivirals (such as remdesivir) as part of a clinical study are eligible for participation in the study (criterion 6)	To allow for enrollment of patients who may receive antivirals as part of a clinical study and receive treatment with investigational therapies under a compassionate use (emergency approval) or expanded access program
5.2 – Exclusion Criteria	Removed verbiage indicating female patients will be evaluated for breastfeeding status or pregnancy at Day 1 (criterion 7).	Correction of a typographical error
5.2 – Exclusion Criteria	Added criterion #9 – Allows enrollment of patients not currently vaccinated against *Neisseria meningitidis* but who will receive prophylactic antibiotic treatment for at least 8 months after last infusion of study drug or until at least 2 weeks after they receive vaccination against *N. meningitidis*	Per Health Authority request
5.4 – Screen Failures	Revised definition	For clarity
6.2 – Preparation/Handling/ Storage/Accountability	Revised instructions to consult the pharmacy manuals	To ensure that investigational sites consult the study Pharmacy Manual for additional details on preparation, handling, storage, and/or accountability
6.4 – Study Intervention Compliance	Removed instructions to consult the pharmacy manual	Patient compliance instructions are not expected to be included in the study Pharmacy Manual

6.5 – Concomitant Therapy	• Added statement that antivirals may be administered as a part of BSC<br>• Added vaccines to the list of examples of concomitant medications that are anticipated to be recorded in the CRF/eCRF	• Per Health Authority request<br>• For clarity
8.3.2.1 – Sequential Organ Failure Assessment Score	• Added statement that the assessment of PaO2 is optional and that the SpO2 will serve as the surrogate for respiratory status<br>• SpO2/FiO2 and the associated cutoff values were added to Table 7<br>• Added footnote to Table 7	To allow for an alternate assessment of the respiratory system to help inform SOFA scoring and for clarity
8.4.1 – Physical Examinations	Revised to indicate that body weight has to be measured, but should be estimated using best judgement if it cannot be measured.	For clarity
8.4.4 – Clinical Safety Laboratory Assessments	Revised to indicate that non-protocol-specified laboratory results that are considered clinically significant are not required to be entered in the CRF/eCRF; however, the Investigator is still required to report (and record in the AE CRF/eCRF) the laboratory abnormality if deemed clinically significant	The study is being implemented in an acute inpatient setting and non-protocol-specified laboratory results may not be readily accessible to sponsor staff for verification.
8.4.8 – Patient Safety Information Card<br>8.4.9 – Vaccine and Antibiotic Prophylaxis	Updated to indicate that the guidance is specific to patients randomized to ravulizumab + BSC	For clarity
8.4.12. Early Termination	Added section	To ensure that patients who are discontinued from the study before Day 29 present for the Early Termination Visit. The patient will also be contacted via telephone on Day 29 to assess health status.
8.5.1 – Clinical Improvement at Day 29	Added section	To better characterize response to treatment
8.5.2 – 12-item Short Form at Days 29, 60, and 90	Added section	To assess impact on health economics
8.5.3 – EuroQol-5 Dimension-5 Level at Days 29, 60, and 90	Added section	To assess impact on health economics
8.8 – Pharmacokinetics<br>8.12 – Immunogenicity<br>10.2 – Appendix 2 (Clinical Laboratory Tests)	Table 9 – Revised to indicate that PK and immunogenicity samples will only be collected from patients randomized to ravulizumab + BSC	To align with the SoA and for clarity
8.9 – Pharmacodynamics	Revised to indicate that PD samples will be collected from all patients	For clarity

8.11 – Biomarkers<br>10.2 – Appendix 2 (Clinical Laboratory Tests)	· Revised to indicate that biomarker samples will be collected from all patients<br>· Revised list of inflammatory and complement pathway biomarkers, and added a coagulation biomarker<br>· Added that biomarkers may also be analyzed by specialty laboratories	For clarity and to ensure the most appropriate samples are collected given the target population and potential underlying diseases
9.1 – Statistical Hypotheses	Reordered list of alternate hypotheses.	To align with the SAP, version 1
9.2 – Sample Size Determination<br>9.5 – Interim Analyses	Revised to indicate that futility was nonbinding	To align with the SAP, version 1
9.4 – Statistical Analyses<br>9.5 – Interim Analyses	Revised to indicate that Primary Evaluation Period and interim analyses will include "available" PK/PD/immunogenicity data	Due to the COVID-19 pandemic, there have been processing delays in collected laboratory samples. Therefore results may not be available in order to perform prespecified statistical analyses.
9.4.1.1 – Analyses of Primary Efficacy Endpoint	· Added process for imputation of missing survival data and specific sensitivity analyses to be conducted for the primary efficacy endpoint (survival [based on all-cause mortality] at Day 29)<br>· Revised the test used for sensitivity analysis of the primary endpoint	To align with the SAP, version 2
9.4.1.2 – Analyses of Primary Efficacy Endpoint	· Revised analyses for the evaluation of selected secondary efficacy endpoints (ie, number of days free of mechanical ventilation at Day 29, duration of ICU stay at Day 29, and SOFA score). Analyses to be conducted for these secondary efficacy endpoints will include a multiple imputation approach using missing at random data. Sensitivity analyses will include the following scenarios: best, worst, and all available. Circumstances under which the analyses will be conducted have also been clarified.<br>· Removed statement that duration of ICU stay will be summarized for nonsurvivors<br>· Revised study day covariate to specify Days 5, 10, 15, 22, and 29 (for SOFA and SpO2/FiO2)	· To align with the SAP, version 2<br>· To align with the SAP, version 2<br>· To align with the SAP, version 1

9.4.1.3 – Multiplicity Adjustments	Revised closed testing order for secondary endpoints. Survival at Day 60 and Day 90 is now excluded from the closed testing procedure, but will be analyzed separately	To align with the SAP, version 1
9.4.2 - Safety Analyses	ECG data was added to the list of safety analyses to be summarized over time.	To align with the SAP, version 1
9.4.3 – Analyses of Pharmacokinetic and Pharmacodynamic	Revised PK and PD analyses	To align with the SAP, version 1
9.4.4 – Analyses of Biomarker	Added that biomarker data will only be summarized for the final study report	To align with the SAP, version 1
9.4.6 – Analyses of Exploratory Endpoint	· Added analyses to be conducted for the new exploratory endpoints of time to clinical improvement<br>· Added analyses for the evaluation of PROs SF-12 and EQ-5D-5L at Days 29, 60, and 90	· To align with the SAP, version 1<br>· PROs were added to evaluate health-related quality of life of patients enrolled in this study
9.5 – Interim Analyses	Revised tests that will be used in the analyses of efficacy and futility.	To align with the SAP, version 2
10.1.6 – Data Quality Assurance	Added clarification that remote source data verification may be employed in the study	Per emerging regulatory guidances
10.2 – Appendix 2 (Clinical Laboratory Tests)	Table 9 – For safety labs to be collected locally; clarified that arterial blood gas to be obtained when available.<br>Added coagulation marker (Factor II); added inflammatory biomarkers (IL-2R, Pentatraxin-3, and Citrullinated histone H3); removed complement pathway biomarkers (C5a, C3a, total C3, Factor B, and Factor Ba)	For clarity and to ensure that the most appropriate biomarker samples relative to the target population are obtained
Throughout	Editorial changes	For clarity, correction of typographical errors, and/or internal document consistency

Abbreviations: AE = adverse event; BSC = best supportive care; C = complement component; COVID-19 = Coronavirus Disease 2019; CRF = case report form; eCRF = electronic case report form; ECG = electrocardiogram; EQ-5D-5L = EuroQol 5-Dimension 5-Level; FiO2 = fraction of inspired oxygen; ICU = intensive care unit; IL = interleukin; IVIg = intravenous immunoglobulin; MCS = Mental Component Summary; PaO2 = partial pressure of oxygen; PCR = polymerase chain reaction; PCS = Physical Component Summary; PD = pharmacodynamic; PK = pharmacokinetic; PRO = patient reported outcomes; SAP = Statistical Analysis Plan; SARS-CoV-2 = severe acute respiratory syndrome coronavirus 2; SF-12 = Short-Form 12; SoA = Schedule of Activities; SOFA = Sequential Organ Failure Assessment; SpO2 = peripheral capillary oxygen saturation.

1. PROTOCOL SUMMARY

1.1. Synopsis

Protocol Title:

A Phase 3 Open-label, Randomized, Controlled Study to Evaluate the Efficacy and Safety of Intravenously Administered Ravulizumab Compared with Best Supportive Care in Patients with COVID-19 Severe Pneumonia, Acute Lung Injury, or Acute Respiratory Distress Syndrome

Short Title:

Efficacy and Safety Study of IV Ravulizumab in Patients with COVID-19 Severe Pneumonia

Rationale:

ULTOMIRIS® (ravulizumab), an effective and widely studied terminal complement inhibitor with a well-established safety profile, is proposed for the treatment of patients who have a confirmed diagnosis of severe acute respiratory syndrome coronavirus 2 (SARS-CoV-2) infection with a clinical presentation consistent with Coronavirus Disease 2019 (COVID-19) severe pneumonia, acute lung injury, or acute respiratory distress syndrome (ARDS). Ravulizumab produces immediate, complete, and sustained inhibition of complement component 5-mediated terminal complement activity. Treatment with ravulizumab could decrease COVID-19-induced lung injury (ie, improve clinical outcomes in patients with COVID-19 severe pneumonia, acute lung injury, or ARDS).

Objectives and Endpoints

Objective	Endpoints
**Primary**	
To evaluate the effect of ravulizumab + BSC compared with BSC alone on the survival of patients with COVID-19	• Survival (based on all-cause mortality) at Day 29
**Secondary**	
To evaluate the efficacy of ravulizumab + BSC compared with BSC alone on outcomes in patients with COVID-19	• Number of days free of mechanical ventilation at Day 29<br>• Duration of intensive care unit stay at Day 29<br>• Change from baseline in SOFA score at Day 29<br>• Change from baseline in SpO2/FiO2 at Day 29<br>• Duration of hospitalization at Day 29<br>• Survival (based on all-cause mortality) at Day 60 and Day 90
**Safety**	
To characterize the overall safety of ravulizumab + BSC compared with BSC alone in patients with COVID-19	• Incidence of TEAEs and TESAEs
**PK/PD/Immunogenicity**	
To characterize the PK/PD and immunogenicity of ravulizumab in patients with COVID-19	• Change in serum ravulizumab concentrations over time<br>• Change in serum free and total C5 concentrations over time<br>• Incidence and titer of anti-ALXN1210 antibodies

Biomarkers	
To assess the effect of C5 inhibition on systemic activation of complement, inflammation, and prothrombic activity in patients with COVID-19	• Change in absolute levels of soluble biomarkers in blood associated with complement activation, inflammatory processes, and hypercoagulable states over time
**Exploratory**	
To evaluate the effect of ravulizumab + BSC compared with BSC alone on progression to renal failure requiring dialysis in patients with COVID-19	• Incidence of progression to renal failure requiring dialysis at Day 29
To evaluate the effect of ravulizumab + BSC compared with BSC alone on clinical improvement in patients with COVID-19	• Time to clinical improvement (based on a modified 6-category ordinal scale) over 29 days
To evaluate the effect of ravulizumab + BSC compared with BSC alone on the health-related quality of life of patients with COVID-19	• SF-12 PCS and MCS scores at Day 29 (or discharge), Day 60, and Day 90
	• EQ-5D-5L scores at Day 29 (or discharge), Day 60, and Day 90

Baseline is defined as the last available assessment on or before Day 1 for all patients. Day 1 will be defined as the date of the first infusion of ravulizumab for patients randomized and dosed with ravulizumab and as the date of randomization for patients randomized but not dosed with ravulizumab.

Abbreviations: BSC = best supportive care; C5 = complement component 5; COVID-19 = Coronavirus Disease 2019; EQ-5D-5L = EuroQol 5-dimension 5-level; FiO2 = fraction of inspired oxygen; MCS = Mental Component Summary; PCS = Physical Component Summary; PD = pharmacodynamic; PK = pharmacokinetic; SF-12 = 12-item Short Form; SOFA = Sequential Organ Failure Assessment; SPO2 = peripheral capillary oxygen saturation; TEAE = treatment-emergent adverse event; TESAE = treatment-emergent serious adverse event.

Overall Design

Study ALXN1210-COV-305 is a multicenter Phase 3, open-label, randomized, controlled study designed to evaluate the safety and efficacy of intravenous (IV) ravulizumab + best supportive care (BSC), compared with BSC alone in patients with a confirmed diagnosis of SARS-CoV-2 infection, and a clinical presentation consistent with COVID-19 severe pneumonia, acute lung injury, or ARDS. Patients at least 18 years of age, weighing ≥ 40 kg, and admitted to a designated hospital facility for treatment will be screened for eligibility in this study. Accounting for a 10% nonevaluable rate, approximately 270 patients will be randomized in a 2:1 ratio (180 patients to receive ravulizumab + BSC, 90 patients to BSC alone).

Patients randomized to ravulizumab + BSC will receive a weight-based dose of ravulizumab on Day 1 (Table s1). On Day 5 and Day 10, doses of 600 mg or 900 mg ravulizumab will be administered (according to weight category) and on Day 15 patients will receive 900 mg ravulizumab. Patients in both treatment groups will continue to receive medications, therapies, and interventions per standard hospital treatment protocols for the duration of the study.

Screening and the Day 1 visits can occur on the same day if the patient has met all inclusion and no exclusion criteria.

Disclosure Statement:

This is an open-label parallel-treatment study.

Number of Patients:

Approximately 270 patients (180 ravulizumab + BSC, 90 BSC alone) will be randomly assigned to 1 of 2 treatment groups.

Intervention Groups and Duration:

The study consists of a Screening Period of up to 3 days, a Primary Evaluation Period of 4 weeks, a final assessment at Day 29, and a Follow-up Period of 8 weeks. The 2 follow-up visits will be conducted 4 weeks apart as a telephone call if the patient is discharged from the hospital or an in-person visit if the patient is still hospitalized. The total duration of each patient's participation is anticipated to be approximately 3 months.

The dosage regimen to be administered during this study is provided in Table s1. No additional doses are allowed during the Primary Evaluation Period (ie, from Day 1 to Day 29).

Table s1: Ravulizumab Dosage Regimen for COVID-19 Severe Pneumonia, Acute Lung Injury, or Acute Respiratory Distress Syndrome

Patient Body Weight (kg)[1]	Day 1 Dose (mg)	Day 5 Dose (mg)	Day 10 Dose (mg)	Day 15 Dose (mg)
40 to < 60	2400	600	600	900
60 to < 100	2700	900	900	900
≥ 100	3000	900	900	900

1. The patient's body weight will be recorded on the day of the infusion visit. If the weight at the day of the infusion cannot be obtained, the weight recorded during the previous study visit may be used.

Abbreviation: COVID-19 = Coronavirus Disease 2019.

Data Monitoring Committee: Yes

1.2. Schema

Figure 1: Study ALXN1210-COV-305 Schematic

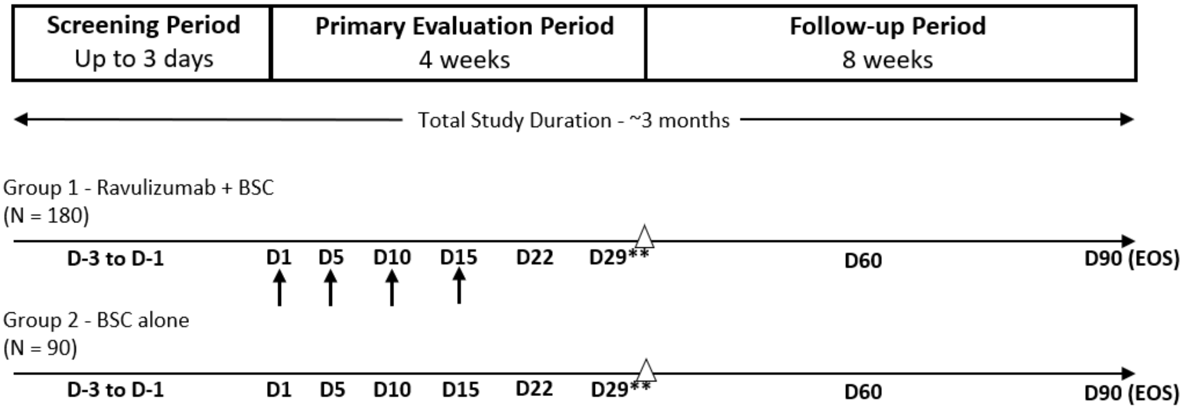

A weight-based dose of ravulizumab will be administered on Day 1 as follows: Patients weighing ≥ 40 to < 60 kg: 2400 mg; ≥ 60 to < 100 kg: 2700 mg; or ≥ 100 kg: 3000 mg.
A weight-based dose of ravulizumab will be administered on Day 5 and Day 10 as follows: Patients weighing ≥ 40 to < 60 kg: 600 mg; ≥ 60 to < 100 kg: 900 mg; or ≥ 100 kg: 900 mg.
On Day 15, patients will receive 900 mg ravulizumab.
**Day 29 represents the end of the Primary Evaluation Period.
Abbreviations: BSC = best supportive care; D = day; EOS = end-of-study; N = number of patients.

1.3. Schedule of Activities

Table 1: Schedule of Activities

**Period**	**Screening Period**		**Primary Evaluation Period**						**Follow-up Period[5]**	
**Study Day (D)**	**D -3 to D -1**	**D1[1,2]**	**D5[2]**	**D10[2]**	**D15[2]**	**D22**	**D29/ Discharge [3]**	**Early Termination[4]**	**D60**	**D90**
**Study Window (days)**	**NA**	**NA**	**±1**	**±1**	**±1**	**±1**	**±1**		**±7**	
**Eligibility**										
Informed consent	X									
Inclusion/exclusion	X									
Medical history[19]	X									
Demographics	X									
**Study Administrative**										
Confirmation of vaccination or appropriate antibiotic prophylaxis against *N. meningitidis*[6]	X								X	
**Screening Laboratory Tests**										
Confirmed positive SARS-CoV-2 test	X									
Chest CT or X-ray[7]	X									
Pregnancy test[8]	X						X	X		
**Randomization**										
Randomized to study drug + BSC or BSC alone		X								
**Administration of Study Drug**										
Ravulizumab[2]		X	X	X	X					
**Efficacy Assessments**										
Survival status		<<monitor continuously>>[18]							X	
Mechanical ventilation status	X	<<monitor continuously>>[18]								
Hospitalization status		<<monitor continuously>>[18]								
Intensive care unit status		<<monitor continuously>>[18]								
Dialysis status		<<monitor continuously>>[18]								
FiO2, SpO2, PaO2[9]		<<monitor daily>>[9]								
Sequential Organ Failure Assessment score	X	X	X	X	X	X	X	X		
**Safety Assessments**										
Electrocardiogram	X						X	X		
Complete physical examination[10]	X						X	X		
Abbreviated physical examination[10]		X	X	X	X	X				
Glasgow Coma Scale	X	X	X	X	X	X	X	X		

**Period**	**Screening Period**		**Primary Evaluation Period**						**Follow-up Period**[5]	
**Study Day (D)**	**D -3 to D -1**	**D1**[1,2]	**D5**[2]	**D10**[2]	**D15**[2]	**D22**	**D29/ Discharge** [3]	**Early Termination**[4]	**D60**	**D90**
**Study Window (days)**	**NA**	**NA**	**±1**	**±1**	**±1**	**±1**	**±1**		**±7**	
Body weight	X	X	X	X	X					
Vital sign measurements (predose)[11]	X	X	X	X	X	X	X	X		
Adverse event review and evaluation	X	<<monitor continuously>>							X	
Review safety card[12]		X[12]					X[12]	X[12]	X[12]	
**Safety Laboratory Tests (Predose)**[13]										
Clinical chemistry	X	X	X	X	X	X	X	X		
Hematology	X	X	X	X	X	X	X	X		
Coagulation panel and D-dimer	X	X	X	X	X	X	X	X		
Urinalysis	X	X	X	X	X	X	X	X		
Direct Coombs test	X									
**PK/PD/Immunogenicity Tests**										
PK[14]		X	X	X	X	X	X	X		
Total and free C5[15]		X	X	X	X	X	X	X		
Immunogenicity (predose)[14]		X					X	X		
**Biomarker Tests**										
Serum and plasma biomarkers (predose)[16]	X	X	X	X	X	X	X	X		
**Other**										
Concomitant medication[17]	X	<<monitor continuously>>							X	
Nonpharmacologic treatments and therapies[18]	X	<<monitor continuously>>							X	
**Patient-reported Outcomes**										
SF-12							X		X	
EQ-5D-5L							X		X	

1. The Day 1 visit may occur on the same day as Screening.
2. Patients randomized to ravulizumab + BSC will receive a weight-based dose of ravulizumab on Day 1 (Table 5). On Day 5 and Day 10, additional doses of 600 mg or 900 mg ravulizumab will be administered (according to weight category) and on Day 15 patients will receive 900 mg of ravulizumab.
3. If a patient is discharged before the end of the Primary Evaluation Period (Day 29), the patient will undergo the early discharge assessments. In addition, the patient will be contacted via telephone on Day 29 to assess health status (eg, survival, mechanical ventilation, hospitalization, intensive care unit, and dialysis). Follow-up telephone calls should be performed as planned unless the patient withdraws consent.
4. The Early Termination Visit is to be conducted when the patient discontinues from the study during the Primary Evaluation Period. In addition, the patient will be contacted via telephone on Day 29 to assess health status (eg, survival, mechanical ventilation, hospitalization, intensive care unit, and dialysis).
5. Additional monitoring will be performed during the 2 follow-up visits to review patient status, including survival and pregnancy, and to obtain information about new or worsening TESAEs. The follow-up will be conducted as a telephone call if the patient is discharged from the hospital or an in-person visit if the patient is still hospitalized.

6. Confirmation of meningococcal vaccination within the past 5 years prior to dosing for patients randomized to ravulizumab + BSC. If vaccination cannot be confirmed, the patient should receive prophylactic antibiotics prior to initiating ravulizumab treatment and for at least 8 months from the last infusion of ravulizumab. When patients are vaccinated less than 2 weeks prior to treatment with ravulizumab or after initiation of ravulizumab, they should continue antibiotic prophylaxis for at least 2 weeks after meningococcal vaccination.
7. Can be performed within the 3 days prior to Screening or at Screening. Imaging performed as part of the patient's routine clinical care is expected and acceptable for inclusion in this study.
8. Urine or serum pregnancy tests (beta human chorionic gonadotropin) to be performed in all female patients. A negative pregnancy test result is required before administration of ravulizumab.
9. SpO2 to be measured by pulse oximetry. PaO2 to be measured by arterial blood gas, if available. FiO2 to be measured by supplemental oxygen. For patients treated with ravulizumab, SpO2, PaO2 (if available), and FiO2 should be measured predose on Day 1. The highest daily measurement of oxygen pressure or saturation on the lowest inspired supplemental oxygen level will be recorded in the CRF/eCRF.
10. Complete or abbreviated physical examination is to be performed at the timepoints indicated in the SoA. A complete physical examination will include, at a minimum, assessments of the following organs/body systems: skin, head, ears, eyes, nose, throat, neck, lymph nodes, chest, heart, abdomen, extremities, and musculoskeletal. An abbreviated physical examination consists of at least an evaluation of the respiratory and cardiovascular systems. Clinically significant abnormalities or findings will be recorded in the AE CRF/eCRF.
11. Vital sign measurements should include systolic and diastolic BP (millimeters of mercury [mm Hg]), heart rate (beats/minute), respiratory rate (breaths/minute), and temperature (degrees Celsius [°C] or degrees Fahrenheit [°F]). These measurements will be taken predose on dosing days.
12. When the patient is responsive and capable of understanding, review the Patient Safety Information Card (including discussion of the risks of meningococcal infections) during the hospitalization and at discharge. Upon discharge, patients who received ravulizumab, must carry the Patient Safety Information Card at all times and for at least 8 months after the last infusion of ravulizumab.
13. Clinical safety laboratory measurements will be collected predose on dosing days.
14. Serum samples for PK and immunogenicity analyses will be collected at the timepoints indicated in the SoA for patients randomized to ravulizumab + BSC. On Day 1/dosing days, immunogenicity and PK samples will be collected within 4 hours before the administration of ravulizumab (predose) and PK samples will be collected within 4 hours after the end-of-infusion (postdose). Postdose PK samples must be collected from a separate line or needle stick to the noninfused arm, not from the infusion line. Pharmacokinetic and immunogenicity samples can be collected at any time on nondosing days during the Primary Evaluation Period.
15. Serum samples for total and free C5 analyses will be collected at the timepoints indicated in the SoA for all patients. For patients randomized to ravulizumab + BSC, samples will be collected within 4 hours before the administration of ravulizumab (predose) and within 4 hours after the end-of-infusion (postdose) on dosing days. Postdose samples must be collected from a separate line or needle stick to the noninfused arm, not from the infusion line. Samples can be collected at any time on nondosing days during the Primary Evaluation Period.
16. Serum and plasma biomarker samples for biomarker analyses will be collected for all patients at the timepoints indicated in the SoA and stored at the investigational site prior to analysis by Alexion or designee. Samples will be collected predose (any time before infusion start) for patients who are randomized to the ravulizumab + BSC treatment group.
17. Concomitant medications and nonpharmacologic therapies considered relevant to the treatment of COVID-19 (BSC) or ravulizumab treatment (eg, antimicrobials, antimalarials, antivirals, steroids, and vasopressors) that the patient is receiving, at the time of Screening and for treating TEAEs/TESAEs, will be recorded in the AE CRF/eCRF.
18. Will be assessed via a telephone call at Day 29 for all patients who are discharged before the end of the Primary Evaluation Period (Day 29).
19. Medical history should also include date of first onset of signs and symptoms of SARS-CoV-2 infection.

Abbreviations: AE = adverse event; BSC = best supportive care; BP = blood pressure; C = complement component/protein; COVID-19 = Coronavirus Disease 2019; CT = computed tomography; D = day; eCRF = electronic case report form; EQ-5D-5L = EuroQol 5-dimension 5-level [questionnaire]

FiO2 = fraction of inspired oxygen; NA = not applicable; PaO2 = partial pressure of oxygen; PD = pharmacodynamics; PK = pharmacokinetics; SARS-COV-2 = severe acute respiratory syndrome coronavirus-2; SF-12 = 12-item Short Form; SoA = Schedule of Activities; SpO2 = peripheral capillary oxygen saturation; TESAE = treatment emergent serious adverse event.

2. INTRODUCTION

2.1. Study Rationale

ULTOMIRIS® (ravulizumab), an effective and widely studied terminal complement inhibitor with a well-established safety profile, is proposed for the treatment of patients who have a confirmed diagnosis of severe acute respiratory syndrome coronavirus 2 (SARS-CoV-2) infection with a clinical presentation consistent with Coronavirus Disease 2019 (COVID-19) severe pneumonia, acute lung injury, or acute respiratory distress syndrome (ARDS). Ravulizumab produces immediate, complete, and sustained inhibition of complement component 5 (C5)-mediated terminal complement activity. Treatment with ravulizumab could decrease COVID-19 induced lung injury (ie, improve clinical outcomes in patients with COVID-19 severe pneumonia, acute lung injury, or ARDS).

2.2. Background

The novel SARS-CoV-2 is a beta-coronavirus identified as the causative agent in COVID-19 (CDC, COVID 19 Situation Summary). Clinical manifestations of COVID-19 range from mild flu-like symptoms (eg, low grade fever, cough, fatigue) to ARDS, respiratory failure, multiple organ failure, and eventual death. An accelerating incidence of SARS-CoV-2 infections have been reported for patients who present with severe pneumonia, acute lung injury, or ARDS.

Emerging epidemiologic data indicate that approximately 30 - 50% of patients with COVID-19 may require hospitalization, approximately 10% may be admitted to critical care units, and 2.5% or more may die of multiorgan failure, especially those individuals who are older or have other comorbidities. For hospitalized patients, the World Health Organization (WHO) has issued recommendations on disease management and therapeutic regimens (WHO, 2020). Aside from supportive care, no therapeutic regimens have been proven effective in reducing either the human-to-human transmission of the infection or its associated fatalities. Mortality in those with critical illness has been reported as > 50%; therefore, implementation of proven critical care interventions such as lung protective ventilation is recommended by WHO.

2.2.1. Nonclinical Evidence Supporting the Role of Complement in COVID-19

Acute respiratory distress syndrome is a constellation of immune-mediated pathologies that are observed in severe cases of coronavirus infection (Hammerschmidt, 1980). This pattern was observed in 2002 with the emergence of severe acute respiratory syndrome-coronavirus (SARS-CoV), and in 2012 when a related coronavirus, Middle East respiratory syndrome coronavirus (MERS-CoV) was identified (Rota, 2003, Zaki, 2012). Complement activation and complement component 5a (C5a; the proinflammatory anaphylatoxin) are involved in multiple mechanisms in the development of acute lung disease induced by pathogenic viruses (Wang, 2015). Emerging evidence suggests that activation of the complement system is involved in the pathogenesis of coronavirus (CoV)-related ARDS, and that a C5 inhibitor may be an effective therapeutic in CoV-mediated disease (Gralinski, 2018).

The complement system is a part of the immune system that enhances the ability of antibodies and phagocytic cells to clear pathogens and damaged cells. It is made up of more than 30 plasma

proteins that opsonize pathogens and induce a series of inflammatory responses to help fight infection. The complement system has key roles in innate and adaptive immune responses, but when hyperactivated can lead to tissue injury. Within the complement system there are 3 pathways (classical, lectin, and alternative) that lead to cleavage of C5 and formation of the membrane attack complex, or terminal complement pathway.

Preclinical data have demonstrated a role for complement activation in CoV-mediated disease. Gralinski evaluated activation of the complement system in a mouse model of CoV. The C57BL/6J mice were infected with mouse-adapted SARS-CoV which resulted in high-titer virus replication within the lung, induction of inflammatory cytokines and chemokines, and immune cell infiltration within the lung. Complement activation was measured by detection of complement pathway component cleavage products. Complement protein 3 activation products were detected in SARS-CoV MA15-infected mice, but not in control mice, as early as 1 day post-infection. Complement protein 3 deposition was observed in the lungs of infected wildtype mice on Day 2 and Day 4 post-infection. Transgenic animals lacking complement component 3 (C3) were protected from SARS-CoV-induced weight loss, had reduced pathology (inflammatory cells in the large airway and parenchyma, perivascular cuffing, thickening of the interstitial membrane, and low levels of intra-alveolar edema), had improved respiratory function, and exhibited lower levels of inflammatory cytokines or chemokines in the lung and periphery. Notably, the kinetics of viral replication were unaltered in the C3-deficient mice relative to wildtype controls, suggesting that the observed effects were due to control of complement-mediated inflammatory processes and not reduction of viral titer. Complement protein 3 is a central hub in the complement cascade and acts as a relay for activation from the alternative pathway. However transgenic mice lacking alternative pathway proteins Factor B or C4 did not have the same protection from CoV-mediated weight loss as compared with C3-deficient mice, suggesting that inhibition of the complement alternative pathway alone is insufficient. This implies that inhibition of a key relay point such as C3 or potentially C5, may be required.

A second model of viral-mediated lung infection also points to a role for complement (Jiang, 2018). A MERS-CoV infection in mice causes severe acute respiratory failure and high mortality accompanied by elevated secretion of cytokines and chemokines. In these infected mice, excessive complement activation was detected. Increased concentrations of C5a and terminal complement complex (C5b-9), activation products resulting from cleavage of C5, were detected in sera and lung tissue, respectively. Blocking C5a with a specific antibody to the C5a receptor (C5aR) reduced alveolar macrophage infiltration and interferon (IFN)-gamma receptor expression in lung, resulting in less tissue damage. Decreased spleen tissue damage was also observed. Interestingly, anti-C5aR treatment led to decreased viral replication in lung tissues.

Patients infected by avian influenza virus H5N1 can also present with severe pneumonia, acute lung injury, or ARDS. The histopathological changes in the lungs are like those observed in severe acute respiratory syndrome (SARS) (Ng, 2006). In a mouse model of H5N1, complement activates immune effector cells and drives lung inflammation. Complement proteins C3a and C5a can increase vascular permeability, recruit and activate leukocytes, activate endothelial cells, upregulate adhesion molecule and cytokine expression, and induce goblet cell secretion of mucus, exacerbating disease through multiple mechanisms. In these mice, deposition of C3, C5b-9, and mannose-binding lectin (MBL)-C was observed in lung tissue. Upregulation of MBL-associated serine protease-2 (MASP-2) and complement receptors C3aR and C5aR was

also detected. Specific inhibition of either C3aR or C5a in the infected mice was effective in reducing lung damage, attenuating inflammation and neutrophil infiltration in the lung, and improving survival (Sun, 2013).

2.2.2. Clinical Hypothesis Supporting Utility of Ravulizumab in COVID-19

Clinical evidence suggests that complement is activated during SARS infection and that the progression of severe pneumonia, acute lung injury, or ARDS in these patients is strongly associated with complement activation. The C3c fragment is present in the sera of patients with SARS and is a strong indicator of disease severity (Pang, 2006). Consistent with this finding, the complement activation product C5a is associated with the inflammatory response and severe lung damage that occurs in patients infected with the 2009 H1N1 influenza virus (Ohta, 2011). It has also been shown that SARS-CoV can directly activate complement via the lectin pathway (Ip, 2005). Patients with SARS develop autoantibodies against human epithelial cells and endothelial cells that mediate complement-dependent cytotoxicity (Yang, 2005).

Multiple lines of evidence support the hypothesis that complement is a key mediator of virally-induced lung damage and that acute lung injury associated with CoV infection is partially mediated by complement (Wong, 2004). Therefore, it is plausible to hypothesize that COVID-19-related injuries and multiorgan failures are mediated, at least in part, by complement activation. The existing data point to the role of C3 and the terminal complement complex, but not the alternative pathway alone. Inhibition of complement, specifically at the terminal complement node through inhibition of C5, may control the inflammatory processes which drive ARDS.

At present, there are no therapies that have received global approval by regulatory authorities for the prevention and/or treatment of COVID-19. China's National Health Commission recently updated treatment guidelines for COVID-19 recommending the use of tocilizumab (anti-interleukin [IL] 6R monoclonal antibody [mAb]) to treat Chinese patients infected with SARS-CoV-2 who have developed serious lung damage and have elevated levels of IL-6 in the blood. A variety of supportive therapies are being used in an attempt to improve prognosis in critically ill patients with confirmed COVID-19 presenting with severe pneumonia, acute lung injury, or potentially life-threatening ARDS. Despite the use of these supportive agents, patients have continued to experience deterioration of respiratory function, a critical contributor to fatal outcomes.

Ravulizumab is a mAb that specifically binds to the complement protein C5 with high affinity, thereby inhibiting its cleavage to C5a and C5b (the initiating subunit of C5b-9) and preventing the generation of the terminal complement complex C5b-9. This mechanism of action provides a therapeutic rationale for the use of ravulizumab in diseases in which complement activation is involved. In addition, the selective blockade of complement cascade at C5 by ravulizumab preserves the activity of upstream components of the complement cascade that are known to be essential for opsonization of microorganisms and prevention of immune complex disorders (Prodinger, 1999). Importantly, C5 blockade preserves the immunoprotective and immunoregulatory functions of early complement components.

Complement inhibition has been shown to be an effective therapeutic target in hematological and neuroinflammatory diseases. Ravulizumab is proposed for the treatment of patients with confirmed SARS-CoV-2 infection with a clinical presentation consistent with COVID-19 severe

pneumonia, acute lung injury, or ARDS. Treatment with ravulizumab produces complete and sustained inhibition of C5-mediated terminal complement activity. Treatment with ravulizumab could improve outcomes in patients with COVID-19 severe pneumonia, acute lung injury, or ARDS.

2.3. Benefit/Risk Assessment

2.3.1. Risk Assessment

Potential risks associated with participation in this study and risk mitigation measures are enumerated in Table 2.

Table 2: Potential Risks and Mitigation Strategies

Risks of Clinical Significance	Summary of Data/ Rationale for Risk	Mitigation Strategy
**Identified risk**		
Meningococcal infection	Patients who have not received a meningococcal vaccination within the past 5 years may be unable to receive meningococcal vaccinations prior to initiating treatment with ravulizumab during this study.	If vaccination cannot be confirmed, the patient should receive prophylactic antibiotics against meningococcal infection prior to initiating ravulizumab treatment and for at least 8 months from the final infusion of ravulizumab (only applicable to patients exposed to study drug).<br><br>When patients are vaccinated less than 2 weeks prior to treatment with ravulizumab or after initiation of ravulizumab, they should continue antibiotic prophylaxis for at least 2 weeks after meningococcal vaccination.
**Potential risks**		
Serious infections	Ravulizumab could increase the risk of infection in this patient population. This potential risk is based on the mode of action of ravulizumab and experience with the use of eculizumab. Since the relevance of serious infection with ravulizumab therapy has not been confirmed in clinical studies, this remains a potential risk.	Training healthcare professionals and patients about the potential risk of additional serious infection. Monitoring for signs and symptoms of serious infections will be conducted as part of routine safety assessments for this study. In addition to appropriate antibiotic coverage versus infection and opportunistic infections, guidelines for immune reconstitution and revaccination will be followed, as applicable.
Immunogenicity	Treatment with any therapeutic protein has the potential to induce an immune response. Potential clinical consequences may include severe hypersensitivity type reactions,	Across 350 patients enrolled in ravulizumab, Phase 3 clinical studies, 2 patients were reported with treatment-emergent ADAs.

Risks of Clinical Significance	Summary of Data/ Rationale for Risk	Mitigation Strategy
	decrease in efficacy and induction of autoimmunity, including antibodies to the endogenous form of the protein (Li, 2002; Casadevall, 2002).	Presence of anti-ALXN1210 antibodies will be assessed.
Infusion site or infusion-associated reactions	Protein therapies administered intravenously have the potential risk of causing local (infusion-site) reactions and systemic reactions (infusion-associated reactions).	Monitoring for infusion reactions will be conducted as part of routine safety assessments for this study. Management of potential infusion reactions is detailed in Section 10.6.
Pregnancy exposure/lactation	No studies of ravulizumab have been conducted in pregnant women. There are no data available on excretion of ravulizumab in breast milk.	Pregnant or nursing female patients will be excluded from participating in this clinical study. Patients and their spouses/partners must use a highly effective or acceptable method of contraception for a period of 8 months following the final infusion of ravulizumab. Breastfeeding should be discontinued during treatment and up to 8 months after the final infusion of ravulizumab.

Abbreviations: ADA = antidrug antibody.

2.3.2. Benefit Assessment

Potential benefits of study participation include:

- Improve survival rate of patients with SARS-CoV-2 infection who are receiving ravulizumab + best supportive care (BSC) compared with BSC alone
- Decrease lung injury in patients with SARS-CoV-2 infection while on supportive medical care
- Improve clinical outcomes in patients with SARS-CoV-2 infection while on supportive medical care

2.3.3. Overall Benefit: Risk Conclusion

Although the efficacy of ravulizumab has not been previously studied in patients with severe pneumonia, acute lung injury, or ARDS, the emerging evidence for the scientific rationale, the predicted drug concentrations and pharmacodynamic (PD) effects after administration of the recommended dose (Section 4.3), and its established safety profile indicate that ravulizumab is an appropriate candidate for clinical investigation, and that the potential for clinical benefit outweighs the risk of treatment with ravulizumab for patients participating in Study ALXN1210-COV-305.

The safety profile of ravulizumab is well characterized in the current clinical development programs, including approved indications in paroxysmal nocturnal hemoglobinuria (PNH) and

atypical hemolytic uremic syndrome (aHUS). Known and potential risks can be effectively managed with the risk mitigation strategies currently in place for ravulizumab.

More detailed information about the known and expected benefits and risks and reasonably expected adverse events (AEs) of ravulizumab may be found in the Investigator's Brochure (IB).

3. OBJECTIVES AND ENDPOINTS

Table 3: Objectives and Endpoints

Objective	Endpoints
**Primary**	
To evaluate the effect of ravulizumab + BSC compared with BSC alone on the survival of patients with COVID-19	• Survival (based on all-cause mortality) at Day 29
**Secondary**	
To evaluate the efficacy of ravulizumab + BSC compared with BSC alone on outcomes in patients with COVID-19	• Number of days free of mechanical ventilation at Day 29<br>• Duration of intensive care unit stay at Day 29<br>• Change from baseline in SOFA score at Day 29<br>• Change from baseline in SpO2/FiO2 at Day 29<br>• Duration of hospitalization at Day 29<br>• Survival (based on all-cause mortality) at Day 60 and Day 90
**Safety**	
To characterize the overall safety of ravulizumab + BSC compared with BSC alone in patients with COVID-19	• Incidence of TEAEs and TESAEs
**PK/PD/Immunogenicity**	
To characterize the PK/PD and immunogenicity of ravulizumab in patients with COVID-19	• Change in serum ravulizumab concentrations over time<br>• Change in serum free and total C5 concentrations over time<br>• Incidence and titer of anti-ALXN1210 antibodies
**Biomarkers**	
To assess the effect of C5 inhibition on systemic activation of complement, inflammation, and prothrombic activity in patients with COVID-19	• Change in absolute levels of soluble biomarkers in blood associated with complement activation, inflammatory processes, and hypercoagulable states over time
**Exploratory**	
To evaluate the effect of ravulizumab + BSC compared with BSC alone on progression to renal failure requiring dialysis in patients with COVID-19	• Incidence of progression to renal failure requiring dialysis at Day 29
To evaluate the effect of ravulizumab + BSC compared with BSC alone on clinical improvement in patients with COVID-19	• Time to clinical improvement (based on a modified 6-point ordinal scale) over 29 days
To evaluate the effect of ravulizumab + BSC compared with BSC alone on the health-related quality of life of patients with COVID-19	• SF-12 PCS and MCS scores at Day 29 (or discharge), Day 60, and Day 90
	• EQ-5D-5L scores at Day 29 (or discharge), Day 60, and Day 90

Baseline is defined as the last available assessment on or before Day 1 for all patients. Day 1 will be defined as the date of the first infusion of ravulizumab for patients randomized and dosed with ravulizumab and as the date of randomization for patients randomized but not dosed with ravulizumab.

Abbreviations: BSC = best supportive care; C5 = complement component 5; COVID-19 = Coronavirus Disease 2019; EQ-5D-5L = EuroQol-5 Dimension-5 level; FiO2 = fraction of inspired oxygen; MCS = Mental Component Summary; PCS = Physical Component Summary; PD = pharmacodynamic; PK = pharmacokinetic; SF-12 = 12-item Short Form; SOFA = Sequential Organ Failure Assessment; SpO2 = peripheral capillary oxygen saturation; TEAE = treatment-emergent adverse event; TESAE = treatment-emergent serious adverse event.

4. STUDY DESIGN

4.1. Overall Design

Study ALXN1210-COV-305 is a multicenter Phase 3, open-label, randomized, controlled study designed to evaluate the safety and efficacy of intravenous (IV) ravulizumab + BSC, compared with BSC alone, in patients with a confirmed diagnosis of SARS-CoV-2 infection, and a clinical presentation consistent with COVID-19 severe pneumonia, acute lung injury, or ARDS. Patients at least 18 years of age, weighing ≥ 40 kg, and admitted to a designated hospital facility for treatment will be screened for eligibility in this study. Accounting for a 10% nonevaluable rate, approximately 270 patients will be randomized in a 2:1 ratio (180 patients to receive ravulizumab + BSC, 90 patients to receive BSC alone).

Patients randomized to ravulizumab + BSC will receive a weight-based dose of ravulizumab on Day 1 (Table s1). On Day 5 and Day 10, doses of 600 mg or 900 mg ravulizumab will be administered (according to weight category) and on Day 15 patients will receive 900 mg ravulizumab. Patients in both treatment groups will continue to receive medications, therapies, and interventions per standard hospital treatment protocols for the duration of the study.

The study consists of a Screening Period of up to 3 days, a Primary Evaluation Period of 4 weeks, a final assessment at Day 29, and a Follow-up Period of 8 weeks. The 2 follow-up visits will be conducted 4 weeks apart as a telephone call if the patient is discharged from the hospital or an in-person visit if the patient is still hospitalized. The total duration of each patient's participation is anticipated to be approximately 3 months (Figure 1).

Screening and the Day 1 visits can occur on the same day if the patient has met all inclusion and no exclusion criteria.

4.2. Scientific Rationale for Study Design

Table 4: Study Design Rationale for Study ALXN1210-COV-305

Design Element	Rationale
This is an open-label, 2:1 randomized, controlled study.	○ A randomized, controlled study design minimizes bias to selection or treatment allocation. This design will ensure identification of an effective treatment that may improve survival in patients with COVID-19 severe pneumonia, acute lung injury, or ARDS.<br>○ The 2:1 randomization will ensure that approximately two-thirds of patients are exposed to treatment and provides more safety information in this patient population for benefit-risk assessment.
Study ALXN1210-COV-305 is being conducted in patients with SARS-CoV-2 infection with a clinical presentation consistent with COVID-19 severe pneumonia, acute lung injury, or ARDS.	○ There is no approved treatment for patients with the severe form of COVID-19.<br>○ Clinical evidence suggests that complement is activated during SARS infection and that the progression of disease is also associated with complement activation.

Design Element	Rationale
	○ Treatment with ravulizumab could 1) decrease lung injury and improve clinical outcomes in patients with SARS-CoV-2 infection while on supportive medical care and 2) provide additional time for patients to recover.
For patients randomized to ravulizumab + BSC, a weight-based dose of ravulizumab will be administered on Day 1. On Day 5 and Day 10, additional doses of 600 mg or 900 mg ravulizumab will be administered (according to weight category) and on Day 15 patients will receive 900 mg ravulizumab.	○ Preliminary PK/PD data suggest that the complement system is amplified in patients with COVID-19 severe pneumonia, acute lung injury, or ARDS beyond what has been observed in patients with aHUS and that additional doses of ravulizumab are needed to provide complete and sustained complement inhibition.
	○ Due to the rapid activation of complement associated with the severe form of COVID-19 (ie, hyperinflammatory response known as cytokine storm syndrome [Mehta, 2020]), this ravulizumab dosage regimen is expected to improve survival and clinically relevant endpoints in these patients.
Duration of study	○ The Primary Evaluation Period was chosen in consideration of the critical status of patients who are admitted to the ICU due to COVID-19 associated lung injury and typical duration of their stay in the ICU.
	○ A Follow-up Period, consisting of 2 follow-up visits conducted approximately 4 weeks apart on Day 60 and Day 90, will be implemented to determine survival, monitor for pregnancy, and obtain information about new or worsening TESAEs. This duration is appropriate given the clinical presentation of COVID-19 patients with lung injury.
Primary endpoint of survival (all-cause mortality) at Day 29	○ The mortality rate of patients with COVID-19 associated severe pneumonia, acute lung injury, or ARDS is staggeringly high (upwards of 50%). Often patients die within a very short time from initial onset of symptoms.<br>○ The chosen primary endpoint is anticipated to reflect a ravulizumab treatment effect over 4 weeks and thus an immediate impact on survival.
Secondary endpoints	○ Most secondary endpoints were selected based on Society of Critical Care Medicine recommendations for standardized endpoints in clinical studies using an intervention to reduce the duration of mechanical ventilation (Blackwood, 2019).<br>○ The SOFA score is a validated endpoint used in the critical care setting to determine clinical outcomes (eg, multiorgan failure) (Lambden, 2019).

Abbreviations: aHUS = atypical hemolytic uremic syndrome; ARDS = acute respiratory distress syndrome; BSC = best supportive care; COVID-19 = Coronavirus Disease 2019; ICU = intensive care unit; PD = pharmacodynamic; PK = pharmacokinetic; SARS = severe acute respiratory syndrome; SARS-CoV-2 = severe acute respiratory syndrome coronavirus 2; SOFA = sequential organ failure assessment; TESAE = treatment-emergent serious adverse event.

4.3. Justification for Dose

CCI [redacted]

[redacted]

[redacted]

[redacted]

CCI [redacted]

CCI [redacted]

CCI

[redacted]

[redacted]

CCI [redacted]

CCI [redacted]	CCI [redacted]
CCI [redacted]	

CCI [redacted]

[redacted]

[redacted]

[redacted]

This is the basis of the therapeutic strategy to achieve complete complement inhibition in patients with COVID-19 infection who present with severe pneumonia, acute lung injury, and/or ARDS.

4.4. End of Primary Evaluation Definition

The end of the Primary Evaluation Period is defined as the date when the last surviving patient completes the Day 29/early termination (ET) visit.

4.5. End of Study Definition

The end of the study is defined as the last patient's last visit, which may be the final safety follow-up telephone call or in-person visit.

5. STUDY POPULATION

Prospective approval of protocol deviations to recruitment and enrollment criteria, also known as protocol waivers or exemptions, are not permitted.

The Sponsor allows a 3-day Screening Period to evaluate patients for eligibility. If the Screening and Day 1 Visits are combined, the patient must meet all inclusion criteria and not meet any exclusion criteria prior to randomization.

5.1. Inclusion Criteria

Patients are eligible to be included in the study only if all the following criteria apply:

Age

1. Patient must be ≥ 18 years of age at the time of providing informed consent.

Type of Patient and Disease Characteristics

2. Confirmed diagnosis of SARS-CoV-2 infection (eg, via polymerase chain reaction [PCR] and/or antibody test) presenting as severe COVID-19 requiring hospitalization.
3. Severe pneumonia, acute lung injury, or ARDS confirmed by computed tomography (CT) or X-ray at Screening or within the 3 days prior to Screening, as part of the patient's routine clinical care.
4. Respiratory distress requiring mechanical ventilation, which can be either invasive (requiring endotracheal intubation) or noninvasive (with continuous positive airway pressure [CPAP] or bilevel positive airway pressure [BiPAP]).

Weight

5. Body weight ≥ 40 kg at the time of providing informed consent.

Sex

6. Male or female.
7. Female patients of childbearing potential and male patients with female partners of childbearing potential must follow protocol-specified contraception guidance for avoiding pregnancy for 8 months after treatment with the study drug (as described in Section 10.4 [Appendix 4]).

5.2. Exclusion Criteria

Patients are excluded from the study if any of the following criteria apply:

Medical Conditions

1. Patient is not expected to survive for more than 24 hours.
2. Patient is on invasive mechanical ventilation with intubation for more than 48 hours prior to Screening.
3. Severe pre-existing cardiac disease (ie, New York Heart Association Class 3 or Class 4, acute coronary syndrome, or persistent ventricular tachyarrhythmias).

4. Patient has an unresolved *Neisseria meningitidis* infection.

Prior/Concomitant Therapy

5. Use of the following medications and therapies:
    a. Current treatment with a complement inhibitor or
    b. Intravenous immunoglobulin (IVIg) within 4 weeks prior to randomization on Day 1.

Prior/Concurrent Clinical Study Experience

6. Treatment with investigational therapy in a clinical study within 30 days before randomization, or within 5 half-lives of that investigational therapy, whichever is greater

    Exceptions:

    a. Investigational therapies will be allowed if received as part of best supportive care through an expanded access protocol or emergency approval for the treatment of COVID-19.
    b. Investigational antiviral therapies (such as remdesivir) will be allowed even if received as part of a clinical study.

Diagnostic Assessments

7. Female patients who are breastfeeding or who have a positive pregnancy test result at Screening.

Other Exclusions

8. History of hypersensitivity to any ingredient contained in the study drug, including hypersensitivity to murine proteins.
9. Patient who is not currently vaccinated against *N. meningitidis*, unless the patient agrees to receive prophylactic treatment with appropriate antibiotics for at least 8 months after the last infusion of study drug or until at least 2 weeks after the patient receives vaccination against *N. meningitidis*.

5.3. Lifestyle Considerations

No lifestyle considerations are applicable for this study.

5.4. Screen Failures

Screen failures are defined as patients who provide informed consent, did not meet any inclusion/exclusion criteria, and are not randomly assigned to treatment.

A minimal set of screen failure information is required to ensure transparent reporting of screen failure patients to meet the Consolidated Standards of Reporting Trials (CONSORT) publishing requirements. Minimal information includes demography (if allowable per local regulations), screen failure details (eg, failed eligibility criteria), and any AEs, including any serious adverse events (SAEs) and any relevant concomitant medication use during the Screening Period.

Individuals who do not meet the criteria for participation in this study (screen failure) due to a reason that is expected to resolve or has resolved may be rescreened based on discussion and agreement between the Investigator and the Medical Monitor.

6. STUDY INTERVENTION

Ravulizumab, a recombinant humanized anti-C5 mAb composed of two 448 amino acid heavy chains and two 214 amino acid light chains, is an IgG2/4 kappa immunoglobulin consisting of human constant regions, and murine complementarity-determining regions grafted onto human framework light- and heavy-chain variable regions. Ravulizumab is produced in Chinese hamster ovarian cell lines and was designed through minimal targeted engineering of eculizumab by introducing 4 unique amino acid substitutions to its heavy chain to extend antibody half-life.

Ravulizumab drug product is supplied for clinical studies as a sterile, preservative-free 10 mg/mL solution in single-use vials and designed for infusion by diluting into commercially available saline (0.9% sodium chloride injection; country-specific pharmacopeia) for administration via IV infusion.

6.1. Study Intervention(s) Administered

The proposed dosage regimen for the treatment of patients with COVID-19 who are ≥ 18 years and ≥ 40 kg and are randomized to ravulizumab + BSC is presented in Table 5.

Table 5: Ravulizumab Dosage Regimen for COVID-19 Severe Pneumonia, Acute Lung Injury, or Acute Respiratory Distress Syndrome

Patient Body Weight (kg)[1]	Day 1 Dose (mg)	Day 5 Dose (mg)	Day 10 Dose (mg)	Day 15 Dose (mg)
40 to < 60	2400	600	600	900
60 to < 100	2700	900	900	900
≥ 100	3000	900	900	900

1. The patient's body weight will be recorded on the day of the infusion visit. If the weight at the day of the infusion cannot be obtained, the weight recorded during the previous study visit may be used.

Abbreviations: COVID-19 = Coronavirus Disease 2019.

Ravulizumab drug product is formulated at pH 7.0 and each 30 mL vial contains 300 mg of ravulizumab, 0.02% polysorbate 80, 150 mM sodium chloride, 6.63 mM sodium phosphate dibasic, 3.34 mM sodium phosphate monobasic, and Water for Injection, United States Pharmacopeia.

The ravulizumab admixture will be administered to the patient using an IV tubing set via an infusion pump followed by an IV flush. Use of a 0.2 micron filter is required during the infusion. The IV flush is infused at the same rate of the infusion and end of flush is considered the end of infusion. The IV flush volume is not to be included in the total volume of study drug administered. Additional details are provided in the Pharmacy Manual.

6.1.1. Packaging and Labeling

Ravulizumab will be manufactured and supplied by Alexion in single 30 mL vials as a solution concentration of 10 mg/mL (Table 6). Each vial contains 300 mg of ravulizumab for IV administration.

Table 6: Ravulizumab Dosage Form and Strength

**Product Name**	Ravulizumab
**Dosage Form**	Concentrate solution for infusion
**Type**	Biologic
**Dose**	300 mg
**NIMP and IMP**	IMP
**Route of Administration**	Intravenous infusion
**Physical Description**	30 mL vial of 10 mg/mL sterile, preservative-free
**Sourcing**	Provided centrally by Alexion or locally by the study site, subsidiary, or designee
**Packaging and Labeling**	Single-use, United States Pharmacopeia/European Pharmacopeia Type 1 clear and colorless glass vial, stoppered with a gray butyl rubber stopper, and sealed with aluminum seal with a polypropylene flip-off cap

Abbreviations: IMP = investigational medicinal product; NIMP = non-investigational medicinal product.

6.2. Preparation/Handling/Storage/Accountability

Stability studies of the diluted admixture of ravulizumab (10 mg/mL) in 0.9% sodium chloride injection support an in-use stability of 6 hours at room temperature at 23°C – 27°C (73°F – 80°F) and 24 hours when refrigerated at 2°C – 8°C (36°F – 46°F).

Ravulizumab vials should not be frozen or shaken.

The Investigator or designee must confirm appropriate temperature conditions have been maintained during transit for all study intervention received and any discrepancies are reported and resolved before use of the study intervention.

Only patients enrolled in the study and randomized to the ravulizumab + BSC group may receive the study intervention and only authorized site staff may supply or administer the study intervention. All study intervention must be stored in a secure, environmentally controlled, and monitored (manual or automated) area in accordance with the labeled storage conditions with access limited to the Investigator and authorized site staff.

The Investigator, institution, or the head of the medical institution (where applicable) is responsible for study intervention accountability, reconciliation, and record maintenance (ie, receipt, reconciliation, and final disposition records).

This responsibility includes the reporting of any product complaints* to PPD within 1 business day of awareness.

**A product complaint is defined as any written, electronic, or oral communication that alleges deficiencies related to the identity, quality, durability, reliability, usability, safety, effectiveness, or performance of a product or clinical study material and/or its packaging components after it is has been released for distribution to an end customer that affects the performance of such product.*

Further guidance and information are provided in the Pharmacy Manual.

6.3. Measures to Minimize Bias: Randomization and Blinding

This is an open-label study. Eligible patients who meet all inclusion and no exclusion criteria will be randomized in a 2:1 ratio to receive either ravulizumab + BSC or BSC alone. Randomization will be stratified by intubated or not intubated on Day 1. A randomization schedule will be developed by a centralized third party.

6.4. Study Intervention Compliance

When patients are dosed at the investigational site, they will receive ravulizumab directly from the Investigator or designee, under medical supervision, thereby minimizing noncompliance. The date and time of the dose administered will be recorded in the source documents and recorded in the case report form (CRF)/electronic case report form (eCRF). The dose of ravulizumab and study patient identification will be confirmed at the time of dosing by a member of the investigational site staff.

6.5. Concomitant Therapy

Patients may receive appropriate concomitant medications, including antivirals, as part of BSC during this clinical study, unless prohibited per exclusion criterion 5.

Concomitant medications considered relevant to treatment of COVID-19 or ravulizumab treatment (eg, vaccines, antimicrobials, antimalarials, antivirals, steroids, and vasopressors) that the patient is receiving at the time of enrollment or receives during the study must be recorded in the CRF/eCRF along with:

- Reason for use,
- Dates of administration, including start and end dates, and
- Dosage information including dose and frequency.

The Medical Monitor should be contacted if there are any questions regarding concomitant therapy.

6.5.1. Disallowed Medicine and Therapy

Use of the following medications and therapies is prohibited for the specified duration prior to Screening and for the duration of the study:

- Current treatment with a complement inhibitor, and
- Intravenous immunoglobulin (IVIg) within 4 weeks prior to randomization on Day 1.

6.6. Dose Modification

Additional doses are not allowed during the study.

6.7. Intervention After the End of the Study

The dosage regimen to be administered during this study is provided in Table 5. Patients will continue to be under the care of their treating physician after the study has concluded.

7. DISCONTINUATION OF STUDY INTERVENTION AND PATIENT DISCONTINUATION/WITHDRAWAL

7.1. Discontinuation of Study Intervention

This is an open-label study. Study drug discontinuation is only applicable for patients who are randomized to ravulizumab + BSC. In rare instances, it may be necessary for a patient to permanently discontinue (definitive discontinuation) the study drug. If the study drug is definitively discontinued, the patient should remain in the study to be evaluated for safety.

Patients should be considered for discontinuation from study drug if any of the following occur:

- Serious hypersensitivity reaction;
- Severe uncontrolled infection;
- Use of disallowed medication as defined in Section 6.5;
- Pregnancy or planned pregnancy; or
- Alexion or the Investigator deems it is necessary for the participant.

Data to be collected at the time of discontinuation of study drug, including follow-up for any further evaluations that need to be completed is provided in the Schedule of Activities (SoA, Table 1).

7.2. Patient Discontinuation/Withdrawal from the Study

- When applicable, all efforts should be made to ensure patients are willing to comply with study participation prior to conducting the screening procedures. The study staff should notify Alexion and their site monitor of all study withdrawals as soon as possible. The reason for patient discontinuation must be recorded in the source documents and CRF/eCRF.
- A patient may withdraw from the study at any time at his/her own request or may be withdrawn at any time at the discretion of the Investigator for safety, behavioral, compliance, or administrative reasons. This is expected to be uncommon.
- At the time of discontinuing from the study, if possible, an ET visit should be conducted. Refer to the SoA (Table 1) for assessments to be collected at the time of study discontinuation and follow-up.
- If the patient withdraws consent for disclosure of future information, Alexion may retain and continue to use any data collected before such a withdrawal of consent.
- If a patient withdraws from the study, he/she may request destruction of any samples taken and not tested, and the Investigator must document this in the site study records.

7.3. Lost to Follow-up

If a patient is unreachable for a scheduled visit within the acceptable visit window (SoA, Table 1), the site study staff must make a reasonable attempt to contact the patient to determine

the reason for missing the visit. If the patient continues to be unreachable, he/she will be considered as lost to follow-up.

Discontinuation of specific sites or of the study as a whole is handled as part of Section 10.1.8.

8. STUDY ASSESSMENTS AND PROCEDURES

- Study procedures and their timing are summarized in the SoA (Table 1). The list of clinical laboratory tests to be performed is provided in Section 10.2.
- Immediate safety concerns should be discussed with Alexion immediately upon occurrence or awareness to determine if the patient should continue or discontinue study intervention.
- Adherence to the study design requirements, including those specified in the SoA (Table 1), is essential and required for study conduct.
- All screening evaluations must be completed and reviewed to confirm that potential patients meet all eligibility criteria. The Investigator will maintain a screening log to record details of all patients screened and to confirm eligibility or record reasons for screening failure, as applicable.
- Procedures conducted as part of the patient's routine clinical management (eg, blood count) and obtained before signing of the informed consent form (ICF) may be utilized for screening or baseline purposes provided the procedures met the protocol-specified criteria and were performed within the time frame defined in the SoA (Section 1.3).

8.1. Eligibility and Administrative Assessments

8.1.1. Informed Consent

Patients or their legally acceptable representative must be consented per the informed consent process outlined in Section 10.1.3. If allowable per local regulations, exceptions may be granted in cases where the patient is unable to provide informed consent.

8.1.2. Inclusion and Exclusion Criteria

All inclusion (Section 5.1) and exclusion (Section 5.2) criteria must be reviewed by the Investigator or qualified designee to ensure the patient qualifies for study participation.

8.1.3. Medical History

The patient's relevant medical history, including prior and concomitant conditions/disorders, treatment history, and history of medical conditions (ie, cardiovascular and respiratory, including smoking status) will be evaluated by the Investigator and documented in the source documents and CRF/eCRF. Medical history should also include date of first onset of signs and symptoms of SARS-CoV-2 infection.

8.1.4. Demographics

A review of demographic parameters, including age, gender, race, and ethnicity will be performed, if allowable per local regulations.

8.1.5. Vaccination or Antibiotic Prophylaxis

Confirmation of meningococcal vaccination within the past 5 years prior to dosing for patients randomized to ravulizumab + BSC. If vaccination cannot be confirmed, the patient should receive prophylactic antibiotics prior to initiating ravulizumab treatment and for at least 8 months from the last infusion of ravulizumab. When patients are vaccinated less than 2 weeks prior to treatment with ravulizumab or after initiation of ravulizumab, they should continue antibiotic prophylaxis for at least 2 weeks after meningococcal vaccination.

Additional guidance is provided in Section 8.4.9.

8.2. Screening Assessments

8.2.1. SARS-COV-2 Testing

The SARS-CoV-2 infection will be evaluated at the designated hospital. A confirmed positive result (eg, via PCR and/or antibody test) is required before randomization.

8.2.2. Chest Computed Tomography or X-ray

Chest CT or X-ray scans will be performed during the Screening Period to confirm findings consistent with severe pneumonia, acute lung injury, or ARDS in patients with COVID-19. Scans performed during the course of the patient's clinical care are accepted and expected to fulfil this diagnostic inclusion criterion for Study ALXN1210-COV-305.

8.2.3. Pregnancy Testing

Urine or serum pregnancy tests (beta human chorionic gonadotropin) will performed in all female patients. A negative pregnancy test result is required before administration of ravulizumab.

8.3. Efficacy Assessments

8.3.1. Primary Efficacy Assessments

Survival at Day 29 will be determined.

8.3.2. Secondary Efficacy Assessments

The following secondary efficacy parameters will also be measured through Day 29:

- Mechanical ventilation status,
- Time in the intensive care unit (ICU),
- Sequential Organ Failure Assessment (SOFA) score,
- Oxygen saturation levels (peripheral capillary oxygen saturation [SpO2]),
- Supplemental oxygen status (fraction of inspired oxygen [FiO2]), and
- Duration of hospitalization.

The following secondary efficacy parameter will be measured at Day 60 and Day 90:

- Survival (based on all-cause mortality)

8.3.2.1. Sequential Organ Failure Assessment Score

Multiple organ failure is a significant indicator of mortality in patients admitted to the ICU. In this study, patients will be evaluated using the SOFA score, an assessment tool that includes a review of 6 organ systems: respiratory, renal, hepatic, cardiac, coagulation, and central nervous system (Vincent, 1998). Each organ system is scored from 0 to 4 points using the worst value observed within the previous 24 hours (Table 7).

Arterial blood gas may not be drawn on a protocol-specified visit day; therefore, the assessment of partial pressure of oxygen (PaO2) is optional and the highly correlated SpO2 will be a surrogate for the respiratory system assessment.

Table 7: Sequential Organ Failure Assessment Scoring

Organ System	Variable (Units)	Score Allocation				
		0	1	2	3	4
Respiratory	PaO2/FiO2 (mmHg)[1]	≥ 400	< 400	< 300	< 200 AND respiratory support (eg, mechanical ventilation)	< 100 AND respiratory support (eg, mechanical ventilation)
	SpO2/FiO2	≥ 302	< 302	< 221	< 142 AND respiratory support (eg, mechanical ventilation)	< 67 AND respiratory support (eg, mechanical ventilation)
Renal	Creatinine (µmol/L)	< 110	110 - 170	171 - 299	300 - 400	> 440
Hepatic	Bilirubin (µmol/L)	< 20	20 - 32	33 - 101	102 - 204	> 204
Cardiac	Inotropes (µg/kg/min)	Mean arterial pressure > 70 mm Hg	Mean arterial pressure < 70 mm Hg	Dopamine ≤ 5 or Dobutamine any dose	Dopamine > 5 or Epinephrine ≤ 0.1 or Norepinephrine ≤ 0.1	Dopamine > 15 or Epinephrine > 0.1 or Norepinephrine > 0.1
Coagulation	Platelets (× 10^3/mm^3)	≥ 150	< 150	< 100	< 50	< 20
CNS	GCS	15	13 - 14	10 - 12	6 - 9	< 6

[1] As arterial blood gas may not be drawn on a protocol-specified visit day, the PaO2 assessment is optional.
Abbreviations: CNS = central nervous system; GCS = Glasgow Coma Scale; FiO2 = fraction of inspired oxygen; PaO2 = partial pressure of oxygen; SpO2 = peripheral capillary oxygen saturation.
Source: Vincent, 1998; Pandharipande, 2006

8.4. Safety Assessments

8.4.1. Physical Examinations

The following safety-related parameters will be measured through Day 29.

- Complete or abbreviated physical examinations will be assessed by the Investigator or designee. A complete physical examination will include, at a minimum, assessments of the skin, head, ears, eyes, nose, throat, neck, lymph nodes, chest, heart, abdomen, extremities, and musculoskeletal. An abbreviated physical examination will include at a minimum, assessment of the respiratory system and cardiovascular systems.
- Body weight should be measured, but if the site does not have the capacity to measure the patient's body weight it should be estimated using best judgement.

Investigators or designees should pay special attention to clinical signs related to previous serious illnesses. Clinically significant abnormalities or findings will be recorded on the AE CRF/eCRF.

8.4.2. Vital Sign Measurements

Vital sign measurements will include systolic and diastolic blood pressure (millimeters of mercury [mm Hg]), heart rate (HR, beats/minute), respiratory rate (RR, breaths/minute), and temperature (degrees Celsius [°C] or degrees Fahrenheit [°F]). Vital sign measurements will be taken predose on dosing days.

8.4.3. Electrocardiograms

- A single 12-lead electrocardiogram (ECG) will be conducted to obtain HR, pulse rate (PR) interval, combination of the Q wave, R wave and S wave (QRS) interval, interval between the start of the Q wave and the end of the T wave (QT), and the corrected QT (QTc) interval(s).

8.4.4. Clinical Safety Laboratory Assessments

- The Investigator must review the laboratory report, document this review, and record any clinically relevant changes occurring during the study in the AE CRF/eCRF. The laboratory reports must be filed with the source documents. Clinically significant abnormal laboratory findings are those that are not associated with the underlying disease, unless judged by the Investigator or designee to be more severe than expected for the patient's condition.
- All laboratory tests with values considered clinically significantly abnormal during participation in the study should be repeated until the values return to normal or baseline or are no longer considered clinically significant by the Investigator or the Medical Monitor.
  - If such values do not return to normal/baseline within a period of time judged reasonable by the Investigator, the etiology should be identified, and Alexion notified.
  - All protocol-required laboratory assessments, as defined in Section 10.2, must be conducted in accordance with the Laboratory Manual and the SoA (Table 1).
  - Laboratory assessments performed at the institution's local laboratory that require a change in patient management or are considered clinically significant by the

Investigator (eg, reported as an SAE or AE), must be recorded in the AE CRF/eCRF.

8.4.5. Immunogenicity

Details of immunogenicity assessments are presented in Section 8.12.

8.4.6. Glasgow Coma Scale

The Glasgow Coma Scale (GCS) is a validated prognostic tool used in the clinical assessment of unconsciousness (eg, patients who are comatose) (Sternbach, 2000).The GCS is comprised of 3 domains – eye response, verbal response, and motor response and within each domain contains a subset of responses that are separately assigned a score (Table 8). The GCS has also been used in the critical care setting as an aid in managing respiratory support. A total GCS score of < 8 is indicative of a patient's need for endotracheal intubation. The GCS will be measured to enable calculation of the secondary efficacy endpoint, SOFA score.

Table 8: Glasgow Coma Scale

Response	Score
**Eye opening**	
Spontaneous	4
To speech	3
To pain	2
None	1
**Best verbal response**	
Oriented	5
Confused conversation	4
Inappropriate words	3
Incomprehensible sounds	2
None	1
**Best motor response**	
Obeys commands	6
Localizes pain	5
Withdrawal (normal flexion)	4
Abnormal flexion (decorticate)	3
Extension (decerebrate)	2
None	1

Source: Sternbach, 2000

8.4.7. Pregnancy

- Pregnancy data from all female patients and female spouses/partners of male patients will be collected from the signing of the ICF until the conclusion of the study participation. If a pregnancy is reported, the Investigator must immediately inform Alexion within 24 hours of awareness of the pregnancy and follow the procedures outlined in Section 10.4.
- For all Alexion products, both in development or post-approval, exposure during pregnancy must be recorded and the pregnancy followed, until the outcome of the pregnancy is known (ie, spontaneous miscarriage, elective termination, normal birth, or

congenital abnormality), even if the patient discontinues the study intervention or withdraws from the study. The corresponding infant must be followed-up with for 3 months postpartum.

- Pregnancy is not considered as an AE (Section 10.4.3) unless there is a suspicion that ravulizumab may have interfered with the effectiveness of a contraceptive medication. However, complications of pregnancy and abnormal outcomes of pregnancy are AEs and may meet the criteria for an SAE (eg, ectopic pregnancy, spontaneous abortion, intrauterine fetal demise, neonatal death, or congenital anomaly) (Section 8.6). Elective abortions without complications should not be reported as AEs.

8.4.8. Patient Safety Information Card

When patients randomized to ravulizumab + BSC are able to understand, a Patient Safety Information Card will be provided to carry with them at all times. The card is provided to increase patient awareness of the risk of meningococcal infections, and promote quick recognition and disclosure of any potential signs or symptoms of infection experienced during the course of the study and to inform patients on what actions must be taken if they are experiencing signs or symptoms of infection.

At each visit throughout the study, the study staff will ensure that the patient has the Patient Safety Information Card. Patients are required to carry the Patient Safety Information Card for 8 months after the last infusion of ravulizumab.

8.4.9. Vaccine and Antibiotic Prophylaxis

It is anticipated that patients randomized to ravulizumab + BSC who have not received a meningococcal vaccination within the past 5 years may be unable to receive meningococcal vaccinations prior to initiating treatment with ravulizumab during this study. If vaccination cannot be confirmed, the patient should receive prophylactic antibiotics against meningococcal infection prior to initiating ravulizumab treatment and for at least 8 months from the last infusion of ravulizumab.

When patients can be vaccinated, vaccines against meningococcal serotypes A, C, Y, W135, and B, where available, are recommended to prevent common pathogenic meningococcal serotypes. Patients must be vaccinated or revaccinated according to the current national vaccination guidelines or local practice for vaccination use with complement inhibitors (eg, ravulizumab). Vaccination may not be sufficient to prevent meningococcal infection. Consideration should be given per official guidance and local practice on the appropriate use of antibacterial agents.

When patients are vaccinated after initiation of ravulizumab, they should continue antibiotic prophylaxis for at least 2 weeks after meningococcal vaccination.

8.4.10. Hospital Discharge

If a patient is discharged before the end of the Primary Evaluation Period, the patient will be contacted via telephone on Day 29 to assess health status (survival, mechanical ventilation, hospitalization, intensive care unit, and dialysis).

8.4.11. Follow-up Monitoring

Follow-up visits will be conducted as indicated in the SoA (Table 1) to review patient status; including survival, monitoring for pregnancy, and to obtain information about new or worsening treatment-emergent SAEs (TESAEs). The follow-up visits will be conducted as a telephone call if the patient is discharged from the hospital or an in person visit if the patient is still hospitalized.

8.4.12. Early Termination

If a patient is discontinued from the study during the Primary Evaluation Period (ie, from Day 1 through Day 29), the patient should present for the Early Termination visit, to be conducted as specified in the SoA (Table 1). The patient will be contacted via telephone on Day 29 to assess health status (eg, survival, mechanical ventilation, hospitalization, intensive care unit, and dialysis).

8.5. Exploratory Assessments

The following exploratory parameters will also be measured:

- Progression to renal failure requiring dialysis at Day 29

8.5.1. Clinical Improvement at Day 29

A reduction in the time to clinical improvement, especially when the patient is treated within a short timeframe from symptom onset has been reported in studies comparing antivirals to placebo (Wang, 2020). Time to clinical improvement will be evaluated during this study and is defined as a live discharge, a decrease from of least 2 points (ie, #5 to #3) from baseline, or both. A modified 6-category ordinal scale (itemized below) will be used to evaluated clinical improvement.

1. Discharged
2. Hospitalized, not requiring supplemental oxygen
3. Hospitalized, requiring supplemental oxygen
4. Hospitalized, requiring noninvasive mechanical ventilation
5. Hospitalized, requiring invasive mechanical ventilation
6. Death

8.5.2. 12-item Short Form at Days 29, 60, and 90

The Short-Form (SF)-12 is a validated health-related quality of life (HR-QoL) instrument that is widely used across a broad spectrum of disease indications. Adapted from the 36 -item SF survey that was designed to evaluate physical and mental health status, the SF-12 survey contains only 12 questions but covers the same 8 domains. There is a further stratification into 2 summary measures (Physical Component Summary [PCS-12] and Mental Component Summary [MCS-12]) as specified below.

- PCS-12

  - General health (1 item)
  - Physical functioning (2 items)
  - Role physical (2 items)
  - Body pain (1 item)
- MCS-12
  - Vitality (1 item)
  - Social Functioning (1 item)
  - Role Emotional (2 items)
  - Mental Health (2 items)

The PCS-12 and MCS-12 summary measures are scored using a norm-based method (ie, mean = 50, SD = 10) (Jenkinson, 1997). A PCS-12 or MCS-12 score of 50 indicates an average score with respect to a healthy population. Scores lower than 50 reflect less than average health and scores greater than 50 reflect better than average health (Ware, 1995).

The SF-12 assumes a recall of 1 week before responding to questions. The survey is anticipated to be completed in several minutes and can be completed by the patient or via an interviewer (in-person or over the telephone).

8.5.3. EuroQol-5 Dimension-5 Level at Days 29, 60, and 90

The EuroQol 5-dimension, 5 severity level (EQ-5D-5L) questionnaire is a brief, validated, HR-QoL instrument that is intended to assess the patient's health status at the time of administration. The questionnaire contains 5 dimensions (mobility, self-care, usual activities, pain/discomfort, and anxiety/depression), each of which includes 5 response variables (no problems, slight problems, moderate problems, severe problems, and extreme problems) (EQ - 5D, 2019) There is no summary score generated upon completion, but rather a 5-digit profile (termed "health state") based on each of the dimensions that can be further converted to a single numerical score (index value).Value sets (a collection of index values) have been derived for multiple countries/regions.

A vertical visual analogue scale (VAS) is included for patients to indicate a self-rated estimate of their health. The VAS ranges from 100 (best health you can imagine) to 0 (worst health you can imagine).

The EQ-5D-5L questionnaire and VAS are anticipated to be completed in several minutes and can be completed by the patient, via an interviewer (in-person or over the telephone); or via proxy.

8.6. Adverse Events and Serious Adverse Events

The definitions of AEs and SAEs can be found in Section 10.3.

All AEs will be reported to the Investigator or qualified designee by the patient (or, when appropriate, by a caregiver, surrogate, or the patient's legally acceptable representative).

The Investigator and any qualified designees are responsible for detecting, documenting, and recording events that meet the definition of an AE or SAE and remain responsible for following up AEs that are serious, considered related to the study intervention or study procedures, or that caused the patient to discontinue the study intervention (see Section 7).

Procedures for recording, evaluating, follow-up, and reporting AEs and SAEs are outlined in Section 10.3 (Appendix 3).

8.6.1. Time Period and Frequency for Collecting AE and SAE Information

All AEs and SAEs will be collected from the time of informed consent until through the timepoints specified in the SoA (Table 1).

All SAEs will be recorded and reported to Alexion or the designee immediately and under no circumstance should this exceed 24 hours, as indicated in Section 10.3 (Appendix 3). The Investigator will submit any updated SAE data to Alexion within 24 hours of it being available.

Investigators are not obligated to actively seek AE or SAE data after conclusion of the study participation. However, if the Investigator learns of any SAE, including a death, at any time after a patient has been discharged from the study, and he/she considers the event to be reasonably related to the study intervention or study participation, the Investigator must promptly notify Alexion.

8.6.2. Method of Detecting AEs and SAEs

The method of recording, evaluating, and assessing causality of AE and SAE and the procedures for completing and transmitting SAE reports are provided in Section 10.3.

Care will be taken not to introduce bias when detecting AEs and/or SAEs. Open-ended and non-leading verbal questioning of the patient is the preferred method to inquire about AE occurrences.

8.6.3. Follow-up of AEs and SAEs

After the initial AE/SAE report, the Investigator is required to proactively follow-up on each patient at subsequent visits/contacts. All SAEs will be followed-up until resolution, stabilization, the event is otherwise explained, or the patient is lost to follow-up (as defined in Section 7.3). Further information on follow-up procedures is provided in Section 10.3.

8.6.4. Regulatory Reporting Requirements for SAEs

- Prompt notification of an SAE by the Investigator to Alexion is essential so that legal obligations and ethical responsibilities towards the safety of patients and the safety of a study intervention under clinical investigation are met.
- Alexion has a legal responsibility to notify both the local regulatory authority and other regulatory agencies about the safety of a study intervention under clinical investigation. Alexion will comply with country-specific regulatory requirements relating to safety reporting to the regulatory authority, Institutional Review Board/Independent Ethics Committee (IRBs/IEC), and Investigators.

- Suspected unexpected serious adverse reactions (SUSARs) must be reported according to local regulatory requirements and Alexion policy and forwarded to Investigators as necessary.
- An Investigator who receives an Investigator safety report describing an SAE or other specific safety information (eg, summary or listing of SAEs) from Alexion will review and then file it along with the IB and will notify the (IRB/IEC), if appropriate according to local requirements.

8.6.5. Adverse Events of Special Interest

Adverse events of special interest that will be monitored during this study are meningococcal infections.

8.7. Treatment of Overdose

No cases of ravulizumab overdose have been reported during clinical studies. Any dose of ravulizumab greater than that specified in the protocol will be considered an overdose.

Overdoses are medication errors that are not considered TEAEs unless there is an untoward medical occurrence resulting from the overdose.

In the event of an overdose, the Investigator or designee should:

1. Contact the Medical Monitor immediately.
2. Closely monitor the patient for any SAE.
3. Obtain a plasma sample for PK analysis if requested by the Medical Monitor (determined on a case-by-case basis).
4. Document the quantity of the excess dose as well as the duration of the overdose in the CRF/eCRF.

8.8. Pharmacokinetics

Samples will be collected from patients randomized to ravulizumab + BSC as specified in the SoA (Table 1) to determine serum concentrations of ravulizumab. The actual date and time (24-hour clock time) of each sample will be recorded.

8.9. Pharmacodynamics

Samples will be collected from all patients as specified in the SoA (Table 1) to assess the effect of ravulizumab on total and free C5 (for patients randomized to ravulizumab + BSC) and determine complement activation in patients randomized to BSC alone. The actual date and time (24-hour clock time) of each sample will be recorded.

8.10. Genetics

Genetics will not be evaluated in this study.

8.11. Biomarkers

Serum and plasma samples will be collected from all patients for biomarker analysis to evaluate complement activation and related pathways and cardiovascular health, and their clinical response to ravulizumab. These biomarkers include complement pathway proteins (eg, total and free C5, soluble C5b-9 [sC5b-9]), cytokines associated with inflammation and disease (eg, IL-1, IL-2R, IL-6, IL-8, IL-21, tumor necrosis factor [TNF]-b, Pentraxin-3, Citrullinated histone H3, and monocyte chemoattractant protein [MCP]-1), Factor II, and markers associated with cardiovascular disease (procalcitonin, myoglobin, high sensitivity troponin I [hs-TnI] and N-terminal pro-b-type natriuretic peptide [NT-proBNP]).

8.12. Immunogenicity

Antibodies to ALXN1210 (ie, antidrug antibody [ADA]) will be evaluated in serum samples collected from patients randomized to ravulizumab + BSC according to the SoA (Table 1). Additionally, serum samples should also be collected at the final visit from patients who discontinued ravulizumab or were withdrawn from the study. These samples will be tested by Alexion or Alexion's designee.

Serum samples will be screened for antibodies binding to ravulizumab and the titer of confirmed positive samples will be reported. Other analyses may be performed to further characterize the immunogenicity of ravulizumab.

The detection and characterization of antibodies to ravulizumab will be performed using a validated assay method. Samples collected for detection of antibodies to ravulizumab will also be evaluated for study intervention serum concentration to enable interpretation of the antibody data. Confirmed antibody positive samples will be further evaluated for antibody titer and the presence of neutralizing antibodies.

8.13. Health Economics Data or Medical Resource Utilization

Data collected during this study that may be used to conduct economic analyses include:

- Duration of hospitalization (total days or length of stay),
- Duration of ICU stay (including total days), and
- Patient reported outcomes (eg, SF-12, version 2 and EQ-5D-5L).

9. STATISTICAL CONSIDERATIONS

9.1. Statistical Hypotheses

The primary null hypothesis is that there is no difference in survival between ravulizumab + BSC and BSC alone as measured by the difference in the proportions surviving at Day 29 between the 2 treatment groups. The alternative hypothesis is that ravulizumab + BSC will improve survival at Day 29 compared with BSC alone.

The null hypotheses associated with the secondary objectives are that ravulizumab + BSC is no different than BSC alone for the respective endpoints; the alternative hypotheses are described below:

1. Number of days free of mechanical ventilation: The alternative hypothesis is that treatment with ravulizumab + BSC will increase the number days free of mechanical ventilation at Day 29 compared with BSC alone.
2. Duration of ICU stay: The alternative hypothesis is that treatment with ravulizumab + BSC will reduce the number days in the ICU at Day 29 compared with BSC alone.
3. Change in SOFA score: The alternative hypothesis is that treatment with ravulizumab + BSC will improve changes in SOFA score at Day 29 compared with BSC alone.
4. Change in SpO2/FiO2: The alternative hypothesis is that treatment with ravulizumab + BSC will improve changes in SpO2/FiO2 at Day 29 compared with BSC alone.
5. Duration of hospitalization: The alternative hypothesis is that treatment with ravulizumab + BSC will reduce the number days in the hospital at Day 29 compared with BSC alone.
6. Survival (based on all-cause mortality) at Day 60 and Day 90: The alternative hypothesis is that ravulizumab + BSC will improve survival at Day 60 and Day 90 compared with BSC alone.

9.2. Sample Size Determination

A sample size of 243 patients (162 ravulizumab + BSC, 81 BSC alone) is required to ensure at least 90% power and detect an improvement in survival from 60% in the BSC alone group to 80% in the ravulizumab + BSC group at Day 29.

This sample size calculation assumes:

- 1-sided Z-test of the difference in 2 proportions,
- Type I error = 0.025,
- Pooled variance,
- 2:1 randomization on the 2 treatment groups,

- One interim analysis at 50% information which will be after collecting primary efficacy data on approximately 122 patients. The early stopping boundaries for efficacy and futility (nonbinding) will be constructed using α-spending function as Lan-DeMets spending function with O'Brien-Fleming flavor and β-spending function as Gamma(-4) (Lan, 1983; Hwang, 1990).

Considering a nonevaluable rate of 10%, this study is planned to randomize approximately 270 patients (180 ravulizumab + BSC, 90 BSC alone).

9.3. Populations for Analyses

The population sets used for analysis sets are defined in the following:

Analysis Set	Description
Intent to Treat (ITT)	The ITT consists of all randomized patients and participants will be analyzed as randomized.<br><br>The ITT will be used for the analysis of efficacy data and is considered the primary analysis population.
Per-Protocol Set (PPS)	The PPS is a subset of the ITT without any important protocol deviations that could impact efficacy analyses. Determination of applicable important protocol deviations for this purpose will be made prior to database lock.<br><br>The PPS will be used for sensitivity analyses of the primary and secondary efficacy endpoints.
Safety Set (SS)	The SS consists of all randomized patients who receive at least 1 dose of ravulizumab for patients randomized to ravulizumab + BSC or who were randomized to BSC alone. The SS will be used for the analysis of safety data.

Abbreviation: BSC = best supportive care.

9.4. Statistical Analyses

The primary analysis will be conducted when all patients have completed the Primary Evaluation Period. This analysis will include all efficacy, safety, and available PK/PD/immunogenicity study data for regulatory submission purposes and will be the final analysis of the Primary Evaluation Period.

Summary statistics will be presented by treatment group and by visit, where applicable. Descriptive statistics for continuous variables will minimally include the number of patients, mean, standard deviation, median, minimum, and maximum. For categorical variables, frequencies and percentages will be presented. Graphical displays will be provided as appropriate. All statistical analyses will be performed based on a 2-sided Type I error of 5%, unless otherwise noted.

Baseline is defined as the last available assessment on or before Day 1 for all patients. Day 1 will be defined as the date of the first infusion of ravulizumab for patients randomized and dosed with ravulizumab and as the date of randomization for patients randomized but not dosed with ravulizumab.

Analyses will be performed using SAS® software Version 9.4 or higher.

9.4.1. Efficacy Analyses

9.4.1.1. Analyses of Primary Efficacy Endpoint

The primary efficacy endpoint is survival (based on all-cause mortality) at Day 29 and will be compared between the 2 treatment groups using a 1-sided Mantel-Haenszel (MH) test of the difference in 2 proportions stratified by intubated or not intubated on Day 1 and a Type I error of 0.025. The estimated MH risk difference will be summarized along with the 95% confidence interval using Mantel-Haenszel stratum weights (Mantel, 1959) and the Sato variance estimator (Sato, 1989). Missing survival data for the primary analysis will be imputed using a multiple imputation approach assuming the data are missing at random (MAR) using a logistic regression model with covariates for treatment group, the randomization stratification factor, age, sex, and presence of a pre-existing condition at baseline. Sensitivity analyses will include the worst-case, all available, and best-case scenarios.

Survival will also be analyzed using the method of Kaplan and Meier (KM) and compared using a log-rank test stratified by intubated or not intubated on Day 1 as a sensitivity analysis. Hazard ratio and risk reduction will be summarized from a Cox proportional hazards model stratified by intubated or not intubated on Day 1. Confidence intervals (95%) will be presented for the survival estimate at Day 29 based on the complementary log-log transformation. Kaplan-Meier curves for both treatment groups will be produced.

A sensitivity analysis of the primary endpoint will also be performed using a 3-level categorical outcome of 3) alive and discharged from the ICU; 2) alive and in the ICU or 1) death. The 2 treatment groups will be compared using an ordinal logistic regression with covariates for treatment group and the randomization stratification factor.

Additional sensitivity analyses will include statistical models adjusting for age, randomization stratification factor, and other important baseline covariates. Subgroup analyses will also be performed by age group, randomization stratification factor, and other important baseline covariates. The Statistical Analysis Plan (SAP) will describe the sensitivity and subgroup analyses in greater detail.

An interim analysis of the primary endpoint will also be conducted as described in Section 9.5.

9.4.1.2. Analyses of Secondary Efficacy Endpoints

Number of days free of mechanical ventilation at Day 29 will be compared between treatment groups using an analysis of covariance (ANCOVA), adjusting for age, and randomization stratification factor, among survivors. Missing data will be imputed using a multiple imputation approach assuming the data are MAR. Sensitivity analyses will include the worst-case, all available, and best-case scenarios.

Duration of ICU stay at Day 29 will be compared between treatment groups using an ANCOVA, adjusting for age and randomization stratification factor, among survivors. Missing data will be imputed using a multiple imputation approach assuming the data are MAR. Sensitivity analyses will include the worst-case, all available, and best-case scenarios.

Changes in SOFA score from Day 1 to Day 29 will be summarized by treatment group and study visit for all patients and will be analyzed using a mixed model for repeated measures (MMRM) with baseline SOFA score, age, randomization stratification factor, treatment group indicator,

study day (Days 5, 10, 15, 22, and 29), and study day by treatment group interaction as covariates. Sensitivity analyses will include imputations for missing data.

Change from baseline in SpO2/FiO2 at Day 29 will be analyzed using a MMRM with baseline SpO2/FiO2, age, randomization stratification factor, treatment group indicator, study day (Days 5, 10, 15, 22, and 29), and study day by treatment group interaction as covariates. All patients will be included in the model. Sensitivity analyses will include imputations for missing data. Change from baseline in PaO2/FiO2 at Day 29 will also be analyzed using a MMRM with baseline PaO2/FiO2, age, randomization stratification factor, treatment group indicator, study day, and study day by treatment group interaction as fixed covariates. All patients will be included in the model. Sensitivity analyses will include imputations for missing data.

Duration of hospitalization at Day 29 will be analyzed in a similar manner as duration of ICU stay.

Survival (based on all-cause mortality) at Day 60 and Day 90 will be estimated using the KM method and compared using a log-rank test stratified by intubated or not intubated on Day 1. Hazard ratio and risk reduction will be summarized from a Cox proportional hazards model stratified by intubated or not intubated on Day 1. Confidence intervals (95%) will be presented for the survival estimates at Day 60 and Day 90 based on the complementary loglog transformation. Kaplan and Meier curves for both treatment groups will be produced.

9.4.1.3. Multiplicity Adjustment

A closed testing procedure will be applied to control the type I error for the analyses of the primary and secondary endpoints. If the primary endpoint is statistically significant in favor of ravulizumab, the secondary endpoints will be evaluated according to the following rank order:

1. Number of days free of mechanical ventilation at Day 29,
2. Duration of ICU stay at Day 29,
3. Change from baseline in SOFA score at Day 29,
4. Change from baseline in SpO2/FiO2 at Day 29,
5. Duration of hospitalization at Day 29.

The hypothesis testing will proceed from highest rank (#1) the number of days free of mechanical ventilation at Day 29 to the lowest rank (#5) duration of hospitalization at Day 29, and if statistical significance is not achieved at an endpoint ($p \geq 0.05$), then endpoints of lower rank will not be considered to be statistically significant. Confidence intervals and p-values will be presented for all secondary efficacy endpoints for descriptive purposes, regardless of the outcome of the closed testing procedure.

An additional secondary endpoint will be assessed beyond Day 29 regardless of the results of the closed testing procedure: Survival (based on all-cause mortality) at Day 60 and Day 90.

9.4.2. Safety Analyses

All safety analyses will be made on the Safety Set (SS). Safety results will be reported by treatment group.

The analysis and reporting of AEs and SAEs will be based on TEAEs and TESAEs, defined as AEs and SAEs with onset during or after treatment with ravulizumab. The incidence of TEAEs and TESAEs will be summarized by System Organ Class and Preferred Term, with additional summaries showing relationship to ravulizumab, severity, TEAEs or TESAEs leading to ravulizumab discontinuation, and TESAEs resulting in death.

Laboratory measurements as well as their changes from baseline at each visit and shift from baseline, if applicable, will be summarized. Vital sign measurements, physical examination findings, and ECG data will also be summarized over time.

9.4.3. Pharmacokinetic and Pharmacodynamic Analyses

All patients who have evaluable PK/PD data will be used to summarize PK/PD parameters for ravulizumab. Descriptive statistics of ravulizumab concentration data will be presented for patients randomized and treated with ravulizumab for each scheduled sampling timepoint.

Total and free C5 concentrations will be evaluated by assessing the absolute values and changes and percentage changes from baseline, as appropriate. Descriptive statistics will be presented by treatment group and for each scheduled sampling timepoint.

9.4.4. Biomarker Analyses

Serum and plasma biomarkers' actual values, and changes from baseline, and their association with observed clinical responses to ravulizumab will be summarized over time, as appropriate. Biomarker data will only be summarized at the final analysis at the end of the study.

9.4.5. Immunogenicity Analyses

The incidence and titers for ADAs to ravulizumab will be summarized in tabular format by treatment group. The proportion of patients ever positive and the proportion of patients always negative may be explored. Confirmed ADA positive samples will be evaluated for the presence of neutralizing antibodies.

9.4.6. Analyses of Exploratory Endpoints

Incidence of and time to progression to renal failure requiring dialysis at Day 29 will be analyzed in a similar manner as the primary endpoint.

Time to clinical improvement will be analyzed using the KM method and compared using a log-rank test stratified by intubated or not intubated on Day 1.

The SF-12 PCS and MCS scores and EQ-5D-5L index and VAS scores will be analyzed using an ANCOVA, adjusting for age and the randomization stratification factor.

9.5. Interim Analyses

An interim analysis for efficacy and futility will be conducted when approximately 122 patients have completed Day 29. If the stopping criteria are met, the study may be terminated early for efficacy or futility depending on which stopping boundary is crossed. The early stopping boundaries for efficacy and futility (nonbinding) will be constructed using α-spending function as Lan-DeMets (O'Brien-Fleming) spending function and β-spending function as Gamma (-4). A

1-sided t-test based on the results from combining all imputed datasets for overall inference will be used with an overall Type I error of 0.025.

The SAP will describe the planned interim analyses in greater detail.

Provided the study was not stopped early for efficacy or futility, the final primary analysis will be conducted when all patients have completed the Primary Evaluation Period. This analysis will include all efficacy, safety, and available PK/PD/immunogenicity study data for regulatory submission purposes. This analysis will not be considered an interim analysis.

9.5.1. Data Monitoring Committee

An independent Data Monitoring Committee (DMC), comprising experts in relevant fields with no direct relationship to the study, will be appointed by Alexion. A minimum of 3 experts (including 1 biostatistician) will be selected. The DMC will review and evaluate cumulative safety data and key efficacy data at prespecified intervals. The DMC’s purview will include recommendations to continue or terminate the study.

Final decisions regarding study conduct will be made by Alexion. Substantive decisions will be communicated to Investigators, IRBs/IECs, and appropriate regulatory authorities.

The specific responsibilities of the DMC including frequency of meetings will be described in the DMC Charter.

10. SUPPORTING DOCUMENTATION AND OPERATIONAL CONSIDERATIONS

10.1. Appendix 1: Regulatory, Ethical, and Study Oversight Considerations

10.1.1. Regulatory and Ethical Considerations

- This study will be conducted in accordance with the protocol and with the following:
  - Consensus ethical principles derived from international guidelines including the Declaration of Helsinki and Council for International Organizations of Medical Sciences (CIOMS) International Ethical Guidelines
  - Applicable International Council for Harmonisation of Technical Requirements for Pharmaceuticals for Human Use (ICH) Good Clinical Practice (GCP) Guidelines
  - Applicable laws and regulations
- The protocol, protocol amendments, ICF, IB, and other relevant documents (eg, advertisements) must be submitted to an IRB/IEC by the Investigator and reviewed and approved by the IRB/IEC before the study is initiated.
- Any amendments to the protocol will require IRB/IEC approval before implementation of changes made to the study design, except for changes necessary to eliminate an immediate hazard to study patients.
- For studies to be approved by Medicines and Healthcare products Regulatory Agency: The Investigator will notify the IRB/IEC of deviations from the study protocol or GCP as defined by UK legislation as a serious breach or as required by IRB/IEC procedures.
- The Investigator will be responsible for the following:
  - Providing written summaries of the status of the study to the IRB/IEC annually or more frequently in accordance with the requirements, policies, and procedures established by the IRB/IEC
  - Notifying the IRB/IEC of SAEs or other significant safety findings as required by IRB/IEC procedures
  - Providing oversight of the conduct of the study at the site and adherence to requirements of 21 CFR, ICH guidelines, the IRB/IEC, European regulation 536/2014 for clinical studies (if applicable), and all other applicable local regulations

10.1.2. Financial Disclosure

Investigators or designees and Sub-Investigators will provide Alexion with sufficient, accurate financial information as requested to allow Alexion to submit complete and accurate financial certification or disclosure statements to the appropriate regulatory authorities. Investigators are

responsible for providing information on financial interests during the course of the study and for 1 year after completion of the study.

10.1.3. Informed Consent Process

- It is the responsibility of the Investigator or designee to obtain informed consent from all patients or their legally acceptable representative as defined per local and country regulations where the study is taking place, and answer all questions regarding the study, prior to any study related procedures including screening assessments.
- Where applicable by national laws and allowed by local regulations, and following IRB/IEC approval, patients who are unable to provide informed consent, and whose legally acceptable representative is unavailable, can be enrolled per the judgement of the Investigator or designee.
- In the exceptional circumstance where informed consent cannot be obtained because of an inability to communicate with, or obtain legally effective consent from the patient and time is not sufficient to obtain consent from the patient's legally acceptable representative, the following procedure should be followed:
  - Written certification from the Investigator and a physician who is not involved with the research must be submitted to the IRB/IEC within 5 working days after administration of the initial dose.
  - If the patient is enrolled without their consent or that of their legally acceptable representative, all reasonable attempts should be made to inform the patient or their legally acceptable representative and/or family of the patient’s enrollment in the study as soon as possible.
  - Document efforts to contact the legally acceptable representative in the study records.
- The Investigator or designee will explain the nature of the study (including but not limited to the objectives, potential benefits and risks, inconveniences, and the patient’s rights and responsibilities) to the patient or his/her legally acceptable representative, defined according to local and country regulations where the study is taking place, and answer all questions regarding the study.
- Patients must be informed that their participation is voluntary. Patients or their legally acceptable representative will be required to sign a statement of informed consent or a certified translation if applicable, that meets the requirements of 21 CFR 50, local regulations, EU General Data Protection Regulation, ICH guidelines, Health Insurance Portability and Accountability Act requirements, where applicable, and the IRB/IEC or study center.
- The patient’s medical record must include a statement that informed consent was obtained before the patient was screened in the study (or as soon as feasible in the case of emergency enrollment by the Investigator or designee) and date the written consent was obtained. The authorized person obtaining the informed consent must also sign the ICF(s).

- Patients must be reconsented to the most current version of the ICFs during their participation in the study.
- A copy of the ICF must be provided to the patient or the patient's legally acceptable representative, as applicable. This document may require translation into the local language. Documentation of ICFs must remain in each patient's study file and must be available for verification at any time.

10.1.4. Data Protection

- Patients will be assigned a unique identifier by Alexion. Any patient records or datasets that are transferred to Alexion will contain the identifier only; patient names or any information which would make the patient identifiable will not be transferred.
- Patients or their legally acceptable representative must be informed that their personal study-related data will be used by Alexion in accordance with local data protection law. The level of disclosure must also be explained to the patients who will be required to give consent for their data to be used as described in the informed consent.
- Patients or their legally acceptable representative must be informed that their medical records may be examined by Clinical Quality Assurance auditors or other authorized personnel appointed by Alexion, by appropriate IRB/IEC members, and by inspectors from regulatory authorities.

10.1.5. Dissemination of Clinical Study Data

Study-related information and study results may be posted on publicly accessible clinical study databases (eg, the US website www.clinicaltrials.gov or the EU website www.clinicaltrialsregister.eu), as appropriate, and in accordance with national, regional, and local regulations.

10.1.6. Data Quality Assurance

- All patient data relating to the study will be recorded on printed or eCRF unless transmitted to Alexion or designee electronically (eg, laboratory data). The Investigator is responsible for verifying that data entries are accurate and correct by physically or electronically signing the eCRF.
- The Investigator must maintain accurate documentation (source data) that supports the information entered in the CRF/eCRF.
- The Investigator must permit study-related monitoring, audits, IRB/IEC review, and regulatory agency inspections and provide direct access to source data documents.
- Alexion or designee is responsible for the data management of this study including quality checking of the data.
- Study monitors will perform ongoing source data verification to confirm that data entered into the CRF/eCRF by authorized site personnel are accurate, complete, and verifiable from source documents; that the safety and rights of patients are being protected; and that the study is being conducted in accordance with the currently

approved protocol and any other study agreements, ICH GCP, and all applicable regulatory requirements.

  - Due to the COVID-19 pandemic, remote source data verification may be employed where permitted by local regulations.
  - The scope of the source data verification will be described in detail in the Monitoring Plan.

- Records and documents, including signed ICFs, pertaining to the conduct of this study must be retained by the Investigator for 2 years after the last marketing application approval, or if not approved, 2 years following the discontinuance of the study intervention, unless local regulations or institutional policies require a longer retention period. No records may be destroyed during the retention period without the written approval of Alexion. No records may be transferred to another location or party without written notification to Alexion.

10.1.7. Source Documents

Source documents provide evidence for the existence of the patient and substantiate the integrity of the data collected. The Investigator or designee will prepare and maintain adequate and accurate source documents (eg, medical records, ECGs, AE and concomitant medication reporting, raw data collection forms) designed to record all observations and other pertinent data for each patient.

Data reported on the CRF/eCRF that are transcribed from source documents must be consistent with the source documents or the discrepancies must be explained. The Investigator may need to request previous medical records or transfer records, depending on the study. Also, current medical records must be available. Source documents are filed at the Investigator's site.

10.1.8. Study and Site Start and Closure

The study start date is the date on which the first patient is consented.

Alexion reserves the right to close the study site or terminate the study at any time for any reason at the sole discretion of Alexion. Study sites will be closed after the study is completed or following the decision to close or terminate the study. A study site is considered closed when all patients have completed the end of study or ET visit, all data have been collected and entered into electronic data capture (EDC) system, all required documents and study supplies have been collected, and a study-site closure visit has been performed.

The Investigator may initiate study-site closure at any time, provided there is reasonable cause and sufficient notice is given in advance of the intended termination.

Reasons for the early closure of a study site by Alexion or Investigator may include but are not limited to:

- Failure of the Investigator to comply with the protocol, the requirements of the IRB/IEC or local health authorities, Alexion's procedures, or GCP guidelines
- Inadequate recruitment of patients by the Investigator
- Discontinuation of further study intervention development

If the study is prematurely terminated or suspended, Alexion shall promptly inform the Investigators, the IRBs/IECs, the regulatory authorities, and any contract research organization(s) used in the study of the reason for termination or suspension, as specified by the applicable regulatory requirements. The Investigator shall promptly inform the patient and should assure appropriate patient therapy and/or follow-up.

10.1.9. Publication Policy

- Where possible, primary manuscripts reporting results of the primary efficacy endpoint or the final results will be submitted for publication within 12 to 18 months of the primary evaluation date or end of study, whichever is earlier.
- Investigators who participate as authors in manuscripts derived from Alexion-sponsored studies will agree to the prerequisites as outlined in the Alexion author engagement agreement prior to engaging in manuscript development.
- The Investigator agrees to submit proposals for new manuscripts (whether or not the proposed analyses are derived from protocol-specified endpoints) to Alexion for review and consideration. All manuscripts or abstracts emanating from approved proposals are to be submitted to Alexion for review before submission to the journal/society. This allows Alexion to protect proprietary information and to provide comments.
  - The proprietary nature of some development work may preclude publication. In some cases, it may be necessary to delay a publication to allow Alexion to ensure protection of intellectual property.
- In general, primary publications, including congress and journal publications, containing the protocol-specified results of a study should occur prior to the publication of individual study site results or case reports. Alexion's policy prohibits duplicate publication, whereby the same results must not be published in multiple peer-reviewed journal manuscripts.
  - Encore congress publications may be appropriate to allow communication of research findings to relevant audience and geographical regions.
- Alexion will comply with the requirements for publication of study results. In accordance with standard editorial and ethical practice, Alexion will generally support publication of multicenter studies only in their entirety and not as individual site data. In this case, a Coordinating Investigator will be designated by mutual agreement.
- Authorship will be determined by mutual agreement and in line with International Committee of Medical Journal Editors authorship requirements and per the Alexion Publication Policy.

10.2. Appendix 2: Clinical Laboratory Tests

- The tests listed in Table 9 may be performed by the local or central laboratory, as appropriate for all patients unless otherwise noted.
- Protocol-specific requirements for inclusion or exclusion of patients are detailed in Section 5 of the protocol.
- Additional tests may be performed at any time during the study as determined necessary by the Investigator or required by local regulations.
- Women of childbearing potential should only be enrolled after a negative serum or urine pregnancy test result at Screening. Additional serum or urine pregnancy testing will be employed as required by site policies, local regulations, or per the requirements of the IRB/IEC and should be performed per the timepoints specified in the SoA (Table 1).
- Investigators must document their review of each laboratory safety report. Clinically significant findings resulting in an assessment of a TESAE should be recorded on the AE CRF/eCRF.

Table 9: Protocol-required Laboratory Assessments

**The following parameters may be analyzed by the local laboratory:**	
**Hematology Panel**<br>Haptoglobin<br>Hematocrit<br>Hemoglobin<br>Platelet count<br>WBC count<br>WBC differential<br><br>**Coagulation Panel**<br>D-dimer<br>International normalized ratio<br>Partial thromboplastin time<br>Prothrombin time<br>Fibrinogen<br><br>**Urinalysis**<br>Albumin<br>Creatinine<br>Protein	**Clinical Chemistry Panel**<br>Alanine aminotransferase<br>Aspartate aminotransferase<br>Blood urea nitrogen<br>C-reactive protein<br>Creatinine<br>Lactate dehydrogenase<br>Sodium<br>Total bilirubin (direct and indirect)<br>Ferritin<br><br>**Other**<br>Arterial blood gas (when available)<br>Beta human chorionic gonadotropin *(all females of childbearing potential only; optional if urine pregnancy test is negative*)<br>Direct Coombs test
**The following parameters may be analyzed by the central laboratory or specialty laboratories:**	

**Inflammatory Biomarkers**<br>IL1, IL-2R, IL-6, IL-8,IL-21, Pentraxin-3, and Citrullinated histone H3<br><br>**Complement Pathway Biomarkers**<br>Total and free C5 and sC5b-9<br><br>**Cardiac Biomarkers**<br>Procalcitonin<br>Myoglobin<br>NT-proBNP<br>hs-TnI	**Other Biomarkers**<br>MCP-1<br>TNF-b<br><br>**Immunogenicity assay**<br>(*only collect from patients randomized to ravulizumab + BSC*)<br><br>**Pharmacokinetic assay**<br>(*only collect from patients randomized to ravulizumab + BSC*)<br><br>**Coagulation Biomarker**<br>Factor II

Abbreviations: BSC = best supportive care; C = complement protein; hs-TnI = high sensitivity troponin I; IL = interleukin, MCP = monocyte chemoattractant protein; NT-proBNP = N-terminal pro b-type natriuretic peptide; sC5b-9 = soluble C5b-9; TNF = tumor necrosis factor; WBC = white blood cell.

10.3. Appendix 3: Adverse Events: Definitions and Procedures for Recording, Evaluating, Follow-up, and Reporting

10.3.1. Definition of AE

**AE Definition**
• An AE is any untoward medical occurrence in a patient, temporally associated with the use of study intervention, whether or not considered related to the study intervention.<br>• Note: An AE can therefore be any unfavorable and unintended sign (including an abnormal laboratory finding), symptom, or disease (new or exacerbated) temporally associated with the use of study intervention.
**Events Meeting the AE Definition**
• Any abnormal laboratory test results (hematology, clinical chemistry, or urinalysis) or other safety assessments (eg, ECG, radiological scans, vital signs measurements), including those that worsen from baseline, considered clinically significant in the medical and scientific judgment of the Investigator (ie, not related to progression of underlying disease).<br>• Exacerbation of a chronic or intermittent pre-existing condition including either an increase in frequency and/or intensity of the condition.<br>• New conditions detected or diagnosed after study intervention administration even though it may have been present before the start of the study.<br>• Signs, symptoms, or the clinical sequelae of a suspected drug-drug interaction.<br>• Signs, symptoms, or the clinical sequelae of a suspected overdose of either study intervention or a concomitant medication. Overdose per se will not be reported as an AE/SAE unless it is an intentional overdose taken with possible suicidal/self-harming intent. Such overdoses should be reported regardless of sequelae.<br>• "Lack of efficacy" or "failure of expected pharmacological action" per se will not be reported as an AE or SAE. Such instances will be captured in the efficacy assessments. However, the signs, symptoms, and/or clinical sequelae resulting from lack of efficacy will be reported as AE or SAE if they fulfill the definition of an AE or SAE.
**Events Not Meeting the AE Definition**
• Medical or surgical procedure (eg, endoscopy, appendectomy): The condition that leads to the procedure is the AE. Situations in which an untoward medical occurrence did not occur (eg, hospitalization for elective surgery if planned before the signing the ICF, admissions for social reasons or for convenience).<br>• Anticipated day-to-day fluctuations of pre-existing disease(s) or condition(s) present or detected at the start of the study that do not worsen.<br>• A medication error (including intentional misuse, abuse, and overdose of the product) or use other than what is defined in the protocol is not considered an AE unless there is an untoward medical occurrence as a result of a medication error.<br>• Cases of pregnancy that occur during maternal or paternal exposure to study intervention are to be reported within 24 hours of Investigator/site awareness. Data on fetal outcome and breastfeeding will be collected for regulatory reporting and safety evaluation.<br>• Any clinically significant abnormal laboratory findings or other abnormal safety assessments which are associated with the underlying disease, unless judged by the Investigator to be more severe than expected for the patient's condition.<br>• The disease/disorder being studied or expected progression, signs, or symptoms of the disease/disorder being studied, unless more severe than expected for the patient's condition.<br>• Situations in which an untoward medical occurrence did not occur (social and/or convenience admission to a hospital).

10.3.2. Definition of SAE

If an event is not an AE per definition above, then it cannot be an SAE even if serious conditions are met (eg, hospitalization for signs/symptoms of the disease under study, death due to progression of disease).

**An SAE is defined as any untoward medical occurrence that, at any dose:**
**1. Results in death**
**2. Is life-threatening**<br>The term "life-threatening" in the definition of "serious" refers to an event in which the patient was at risk of death at the time of the event. It does not refer to an event, which hypothetically might have caused death, if it was more severe.
**3. Requires inpatient hospitalization or prolongation of existing hospitalization**<br>In general, hospitalization signifies that the patient has been detained (usually involving at least an overnight stay) at the hospital or emergency ward for observation and/or treatment that would not have been appropriate in the physician's office or outpatient setting. Complications that occur during hospitalization are AEs. If a complication prolongs hospitalization or fulfills any other serious criteria, the event is serious. When in doubt as to whether "hospitalization" occurred or was necessary, the AE should be considered serious.<br>Hospitalization for elective treatment of a pre-existing condition that did not worsen from baseline is not considered an AE.
**4. Results in persistent disability/incapacity**<br>• The term disability means a substantial disruption of a person's ability to conduct normal life functions.<br>• This definition is not intended to include experiences of relatively minor medical significance such as uncomplicated headache, nausea, vomiting, diarrhea, influenza, and accidental trauma (eg, sprained ankle) which may interfere with or prevent everyday life functions but do not constitute a substantial disruption.
**5. Is a congenital anomaly/birth defect**
**6. Other situations:**<br>• Medical or scientific judgment should be exercised in deciding whether SAE reporting is appropriate in other situations such as important medical events that may not be immediately life-threatening or result in death or hospitalization but may jeopardize the patient or may require medical or surgical intervention to prevent one of the other outcomes listed in the above definition. These events should usually be considered serious.<br>• Examples of such events include invasive or malignant cancers, intensive treatment in an emergency room or at home for allergic bronchospasm, blood dyscrasias or convulsions that do not result in hospitalization, or development of drug dependency or drug abuse.

10.3.3. Recording and Follow-Up of AE and/or SAE

**Recording of AE and/or SAE**
• When an AE/SAE occurs, it is the responsibility of the Investigator to review all documentation (eg, hospital progress notes, laboratory reports, and diagnostics reports) related to the event.<br>• The Investigator will then record all relevant AE/SAE information in the CRF/eCRF.<br>• It is not acceptable for the Investigator to send photocopies of the patient's medical records to Alexion in lieu of completion of the Alexion/AE/SAE CRF/eCRF page.<br>• There may be instances when copies of medical records for certain cases are requested by Alexion. In this case, all patient identifiers, with the exception of the patient number, will be redacted on the copies of the medical records before submission to Alexion.<br>• The Investigator will attempt to establish a diagnosis of the event based on signs, symptoms, and/or other clinical information. Whenever possible, the diagnosis (not the individual signs/symptoms) will be documented as the AE/SAE.
**Assessment of Intensity**
The Investigator will make an assessment of intensity for each AE and SAE reported during the study and assign it to one of the following categories from National Cancer Institute CTCAE v5.0, published 27 Nov 2017:<br>• Grade 1: Mild (awareness of sign or symptom, but easily tolerated)<br>• Grade 2: Moderate (discomfort sufficient to cause interference with normal activities)

- Grade 3: Severe (incapacitating, with inability to perform normal activities)
- Grade 4: Life-threatening
- Grade 5: Fatal
- An event is defined as “serious” when it meets at least one of the predefined outcomes as described in the definition of an SAE, not when it is rated as severe.

Assessment of Causality

- The Investigator is obligated to assess the relationship between the study intervention and each occurrence of each AE or SAE. An Investigator causality assessment must be provided for all AEs (both nonserious and serious). This assessment must be recorded in the CRF/eCRF and on any additional forms, as appropriate. The definitions for the causality assessments are as follows:
  - Not related: There is no reasonable possibility the study intervention caused the AE.
    - The AE has a more likely alternative etiology; it may be due to underlying or concurrent illness, complications, concurrent treatments, or effects of another concurrent drug.
    - The event does not follow a reasonable temporal relationship to administration of the study intervention.
  - Related: There is a reasonable possibility the study intervention caused the AE.
    - The AE has a temporal relationship to the administration of the study intervention.
    - The event does not have a likely alternative etiology.
    - The event corresponds with the known pharmaceutical profile of the study intervention.
    - There is improvement on discontinuation and/or reappearance on rechallenge.
- The Investigator will use clinical judgment to determine the relationship.
- Alternative causes, such as underlying disease(s), concomitant therapy, and other risk factors, as well as the temporal relationship of the event to study intervention administration will be considered and investigated.
- The Investigator will also consult the IB and/or Product Information, for marketed products, in his/her assessment.
- For each AE/SAE, the Investigator **must** document in the medical notes that he/she has reviewed the AE/SAE and has provided an assessment of causality.
- There may be situations in which an SAE has occurred, and the Investigator has minimal information to include in the initial report to Alexion. However, it is very important that the Investigator always make an assessment of causality for every event before the initial transmission of the SAE data to Alexion.
- The Investigator may change his/her opinion of causality in light of follow-up information and send an SAE follow-up report with the updated causality assessment.
- The causality assessment is one of the criteria used when determining regulatory reporting requirements.

Follow-up of AEs and SAEs

- The Investigator is obligated to perform or arrange for the conduct of supplemental measurements and/or evaluations as medically indicated or as requested by Alexion to elucidate the nature and/or causality of the AE or SAE as fully as possible. This may include additional laboratory tests or investigations, histopathological examinations, or consultation with other health care professionals.
- New or updated information will be recorded in the originally completed CRF/eCRF.
- The Investigator will submit any updated SAE data to Alexion within 24 hours of receipt of the information.

10.3.4. Reporting of SAEs

SAE Reporting to Alexion via an Electronic Data Collection Tool

- All SAEs will be recorded and reported to Alexion or designee immediately and within 24 hours of awareness.
- The primary mechanism for reporting an SAE to Alexion will be the electronic data collection tool.
- If the electronic system is unavailable at the time that the Investigator or site becomes aware of an SAE, the site will use the paper Contingency Form for SAE reporting via fax or email. Facsimile transmission or email may be used in the event of electronic submission failure.
  - Email: PPD or Fax: PPD
- The site will enter the SAE data into the EDC system as soon as it becomes available.

- When further information becomes available, the EDC should be updated within 24 hours with the new information and an updated SAE report should be submitted to Alexion global drug safety (GDS).
- After the study is completed at a given site, the electronic data collection tool will be taken off-line to prevent the entry of new data or changes to existing data.
- If a site receives a report of a new SAE from a study patient or receives updated data on a previously reported SAE after the electronic data collection tool has been taken off-line, then the site can report this information on a paper SAE form (see next section) or to the Alexion/Medical Monitor/SAE Coordinator by telephone.

SAE Reporting to Alexion via Paper Safety Reporting Form

- All SAEs will be recorded and reported to Alexion or designee immediately and within 24 hours awareness.
- SAEs will be reported using the Safety Reporting Form and submitted to Alexion GDS. The Investigator must complete, sign, and date the SAE pages, verify the accuracy of the information recorded on the SAE pages with the corresponding source documents, and send a copy via email or facsimile to the contact information provided below:
    - Email: PPD or Fax: PPD
- Additional follow-up information, if required or available, should be entered into the CRF/eCRF and sent to Alexion GDS within 24 hours of the Investigator or study site staff becoming aware of this additional information via the reporting process outlined above.
- For all SAEs, the Investigator must provide the following:
    - Appropriate and requested follow-up information in the time frame detailed above
    - Causality of the SAE(s)
    - Treatment of/intervention for the SAE(s)
    - Outcome of the SAE(s)
    - Medical records and laboratory/diagnostic information
- All paper forms and follow-up information submitted to Alexion GDS **must** be accompanied by a cover page signed by the Investigator.
- Paper source documents and/or reports should be kept in the appropriate section of the study file.

10.4. Appendix 4: Contraceptive Guidance and Collection of Pregnancy Information

10.4.1. Definitions

Woman of Childbearing Potential (WOCBP)

A woman is considered fertile following menarche and until becoming postmenopausal unless permanently sterile (see below).

If fertility is unclear (eg, amenorrhea in adolescents or athletes) and a menstrual cycle cannot be confirmed before administering the dose of study intervention, additional evaluation should be considered.

Women in the following categories are not considered WOCBP

1. Premenarchal
2. Premenopausal female with one of the following:
   - Documented hysterectomy
   - Documented bilateral salpingectomy
   - Documented bilateral oophorectomy
   - For individuals with permanent infertility due to an alternate medical cause other than the above, (eg, Mullerian agenesis, androgen insensitivity), Investigator discretion should be applied to determining study entry.
   - Note: Documentation can come from the site personnel's: review of the patient's medical records, medical examination, or medical history interview.
3. Postmenopausal female
   - A postmenopausal state is defined as no menses for 12 months without an alternative medical cause.
     - A high follicle stimulating hormone (FSH) level in the postmenopausal range may be used to confirm a postmenopausal state in women not using hormonal contraception or hormonal replacement therapy (HRT). However, in the absence of 12 months of amenorrhea, confirmation with more than 1 FSH measurement is required.
   - Females on HRT and whose menopausal status is in doubt will be required to use one of the non-estrogen hormonal highly effective contraception methods if they wish to continue their HRT during the study. Otherwise, they must discontinue HRT to allow confirmation of postmenopausal status before study enrollment.

10.4.2. Contraception Guidance

10.4.2.1. Guidance for Female Patients

Female patients of non-childbearing potential are exempt from contraception requirements. Non-childbearing potential for female patients is defined as any of the following:

1. Prior to first menses
2. Postmenopausal, as documented by amenorrhea for at least 1 year prior to the Day 1 visit and FSH serum levels consistent with postmenopausal status
3. Permanent sterilization at least 6 weeks prior to the Day 1 visit:
4. Hysteroscopic sterilization
5. Bilateral tubal ligation or bilateral salpingectomy
6. Hysterectomy
7. Bilateral oophorectomy

Female patients of childbearing potential must use a highly effective method of contraception, including at least one of the following:

1. Intrauterine device (without copper) in place for at least 6 weeks.
2. Progestogen-only hormonal contraception (either oral, injectable, or implantable) for at least 6 weeks.
3. Intrauterine progestogen releasing system for at least 6 weeks.
4. Bilateral tubal occlusion for at least 6 weeks.
5. Combined (estrogen- and progestogen-containing) hormonal contraception (either oral, intravaginal, or transdermal) for at least 6 weeks. Estrogen-containing hormonal contraception is acceptable only if it has been used for at least 6 weeks immediately prior to the Day 1 visit. Estrogen-containing hormonal contraception may not be initiated during the study period.
6. Surgical sterilization of the male partner (medical assessment of azoospermia is required if vasectomy was performed within the prior 6 months).
7. Sexual abstinence for female patients:
   a. Sexual abstinence is considered a highly effective method only if defined as refraining from heterosexual intercourse. In this study, abstinence is only acceptable if consistent with the patient’s preferred and usual lifestyle. Abstinent female patients who wish to initiate a highly effective method of contraception during the study must refrain from heterosexual intercourse for at least 1 menstrual cycle.
   b. Periodic abstinence (eg, calendar, symptothermal, or post-ovulation methods) is not considered a highly effective method of contraception for female patients.

Other methods of contraception that are not considered highly effective for female patients:

1. Barrier methods, such as male or female condoms, diaphragm, or cervical cap, used alone or in combination, are not acceptable.
2. Spermicides or spermicidal sponges, used alone or in combination with barrier methods, are not acceptable.

Withdrawal (*coitus interruptus*) is not acceptable.

Lactational amenorrhea is not acceptable.

Female patients randomized to ravulizumab + BSC must not donate ova from the Day 1 visit until 8 months after treatment with the last infusion of study drug.

10.4.2.2. Guidance for Male Patients

Contraception is the responsibility of the heterosexually active male patients, regardless of his female partner's method of contraception.

Male patients who have had a vasectomy > 6 months prior must use a condom during heterosexual intercourse. Male patients who have had a vasectomy < 6 months prior must use a condom and spermicide during heterosexual intercourse.

Male patients who have not had a vasectomy must use a condom and spermicide during heterosexual intercourse from the Day 1 visit until 8 months after treatment with the last infusion of study drug.

10.4.2.2.1. Sexual Abstinence for Male Patients

Sexual abstinence is considered a highly effective method only if defined as refraining from heterosexual intercourse. In this study, abstinence is only acceptable if consistent with the patients' s preferred and usual lifestyle. Abstinent male patients who become heterosexually active must use a condom and spermicide during intercourse.

Periodic abstinence (eg, calendar, symptothermal, or post-ovulation methods for a female partner) is not considered a highly effective method of contraception for male patients.

Male patients randomized to ravulizumab + BSC must not donate sperm from the Day 1 visit until 8 months after treatment with the last infusion of study drug.

10.4.3. Collection of Pregnancy Information

Pregnancy data will be collected during this study for all female patients and female spouses/partners of male patients. Exposure during pregnancy (also referred to as exposure in utero) can be the result of either maternal exposure or transmission of study intervention via semen following paternal exposure. If a female patient or a male patient's female partner becomes pregnant during the conduct of this study, the Investigator must submit the "Pregnancy Reporting and Outcome/Breastfeeding" form to Alexion GDS via facsimile or email. When the outcome of the pregnancy becomes known, the form should be updated and submitted to Alexion GDS. If additional follow-up is required, the Investigator will be requested to provide the information.

Exposure of an infant to study intervention during breastfeeding must also be reported (via the "Pregnancy Reporting and Outcome Form/Breastfeeding") and any AEs experienced by the infant must be reported to Alexion GDS or designee via email or facsimile.

Pregnancy is not regarded as an AE unless there is a suspicion that the study intervention may have interfered with the effectiveness of a contraceptive medication. However, complications of pregnancy and abnormal outcomes of pregnancy are AEs and may meet the criteria for an SAE (eg, ectopic pregnancy, spontaneous abortion, intrauterine fetal demise, neonatal death, or congenital anomaly). Elective abortions without complications should not be reported as AEs.

Any female patient who becomes pregnant while participating in the study will be discontinued from study intervention.

10.4.3.1. Male Patients With Partners Who Become Pregnant

- The Investigator will attempt to collect pregnancy information on any male patient's female partner who becomes pregnant while the male patient is in this study. This applies only to male patients who receive ravulizumab.
- After obtaining the necessary signed informed consent from the pregnant female partner directly, the Investigator will record pregnancy information on the appropriate Pregnancy Outcome/Breastfeeding form and submit it to Alexion within 24 hours of learning of the partner's pregnancy. The female partner will also be followed to determine the outcome of the pregnancy. Information on the status of the mother and child will be forwarded to Alexion. Generally, the follow-up will be no longer than 6 to 8 weeks following the estimated delivery date. Any termination of the pregnancy will be reported regardless of fetal status (presence or absence of anomalies) or indication for the procedure.

10.4.3.2. Female Patients Who Become Pregnant

- The Investigator will collect pregnancy information on any female patient who becomes pregnant while participating in this study. The initial Information will be recorded on the appropriate form and submitted to Alexion within 24 hours of learning of a patient's pregnancy.
- The patient will be followed-up to determine the outcome of the pregnancy. The Investigator will collect follow-up information on the patient and the neonate and the information will be forwarded to Alexion. Generally, follow-up will not be required for longer than 3 months beyond the estimated delivery date. Any termination of pregnancy will be reported, regardless of fetal status (presence or absence of anomalies) or indication for the procedure.
- While pregnancy itself is not considered to be an AE or SAE, any pregnancy complication or elective termination of a pregnancy for medical reasons will be reported as an AE or SAE. A spontaneous abortion (occurring at < 22 weeks gestational age) or still birth (occurring at > 22 weeks gestational age) is always considered to be an SAE and will be reported as such. Any post-study pregnancy related SAE considered reasonably related to the study intervention by the Investigator will be reported to Alexion as described in Section 8.6.4. While the Investigator is not obligated to actively seek this information in former study patients, he or she may learn of an SAE through spontaneous reporting.

10.5. Appendix 5: Biomarkers

- Blood samples will be collected for biomarker analyses and the data may be used for future exploratory research related to complement activation and inflammatory processes. The samples may also be used to develop tests/assays including diagnostic tests related to C5 inhibitors and COVID-19 with clinical presentation of severe pneumonia, acute lung injury, or ARDS.
- The samples may be analyzed as part of a multistudy assessment of biomarkers in the response to ravulizumab to understand COVID-19 or related conditions.
- The results of biomarker analyses may be reported in a final clinical study report or in a separate summary report.
- Alexion or designee will store the samples obtained for biomarker analyses in a secure storage space with adequate measures to protect confidentiality.

10.6. Appendix 6: Management of Potential Drug Infusion Reactions

Intravenous and infusion-associated reactions are a potential risk with the use of monoclonal antibodies; these reactions can be nonimmune or immune mediated (eg, hypersensitivity reactions). Signs and symptoms may include headache, fever, facial flushing, pruritus, myalgia, nausea, chest tightness, dyspnea, vomiting, erythema, abdominal discomfort, diaphoresis, shivers, hypertension, lightheadedness, hypotension, palpitations, and somnolence. Signs and symptoms of hypersensitivity or allergic reactions may include hives, swollen face, eyelids, lips, or tongue, or trouble with breathing.

All administration-, IV-, and infusion-associated reactions will be reported to the Investigator and qualified designee. The Investigator and qualified designee are responsible for detecting, documenting, and recording events that meet the definition of AE or SAE and remain responsible for following up events that are serious, considered related to the study drug, or study procedures; or that caused the participant to discontinue ravulizumab (Section 7).

Definitions and procedures for recording, evaluating, follow-up, and reporting AEs and SAEs are outlined in Section 10.3.

Before any infusion is started, the treating physician and other appropriate personnel must make certain that medication (ie, adrenaline, inhaled beta agonists, antihistamines, corticosteroids) and other equipment to treat anaphylaxis are readily available. The infusion must be stopped immediately if Grade ≥ 2 allergic/hypersensitivity reactions (including drug fever) or Grade ≥ 3 cytokine release syndrome/acute infusion reaction occurs. The Sponsor must be notified within 24 hours of any infusion reaction requiring interruption or discontinuation of study drug.

Patients who experience a reaction during the administration of study drug should be treated according to institutional guidelines. For a Grade 1 or Grade 2 infusion reaction, the infusion should be temporarily stopped and treatment with an antihistamine (eg, diphenhydramine 25 to 50 mg orally or equivalent) and acetaminophen (650 mg orally or equivalent) may be considered. If the patient's signs and symptoms have resolved (with or without administration of the above medication), the infusion may be restarted. However, the patients should be infused at a slower rate and be monitored closely for any signs and symptoms of infusion reactions during the remainder of the infusion. Patients experiencing an infusion reaction should be observed in the clinic until resolution of the reaction, or until the Investigator determines the patient is no longer at risk. Patients who experience a severe reaction during administration of study drug resulting in discontinuation of study drug should undergo all scheduled safety, PK, and PD evaluations required by the protocol.

If anaphylaxis occurs according to the criteria listed below, then administration of subcutaneous epinephrine (1/1000, 0.3 mL to 0.5 mL, or equivalent) should be considered. In the case of bronchospasm, treatment with an inhaled beta agonist also should be considered. Patients administered an antihistamine for the treatment or prevention of an infusion reaction should be given appropriate warnings about drowsiness and impairment of driving ability before being discharged from the center.

Table 10: Clinical Criteria for Diagnosing Anaphylaxis

**Anaphylaxis is highly likely when any 1 of the following 3 criteria is fulfilled:**
• Acute onset of an illness (minutes to several hours) with involvement of the skin, mucosal tissue, or both (eg, generalized hives, pruritus or flushing, swollen lips-tongue-uvula), and at least 1 of the following:<br>○ Respiratory compromise (eg, dyspnea, wheeze-bronchospasm, stridor, reduced peak expiratory flow, hypoxemia)<br>○ Reduced blood pressure or associated symptoms of end-organ dysfunction (eg, hypotonia [collapse], syncope, incontinence)
• Two or more of the following that occur rapidly after exposure to a likely allergen for that participant (minutes to several hours):<br>○ Involvement of the skin-mucosal tissue (eg, generalized hives, itch-flush, swollen lips/tongue/uvula)<br>○ Respiratory compromise (eg, dyspnea, wheeze/bronchospasm, stridor, reduced peak expiratory flow, hypoxemia)<br>○ Reduced blood pressure or associated symptoms (eg, hypotonia [collapse], syncope, incontinence)<br>○ Persistent gastrointestinal symptoms (eg, crampy abdominal pain, vomiting)
• Reduced blood pressure after exposure to known allergen for that participant (minutes to several hours):<br>○ Systolic blood pressure of less than 90 mmHg or greater than 30% decrease from that participant's baseline

Source: (Sampson, 2006)

10.7. Appendix 7: Protocol Amendment History

The Protocol Amendment Summary of Changes table for the current amendment is located directly before the Table of Contents.

**DOCUMENT HISTORY**		
**Document**	**Date**	**Summary of Key Changes in the Amendment**
Amendment 2.3 (France)	08 May 2020	• Updates implemented in this protocol amendment were based on Ethics Committee feedback.<br>o Added specific informed consent measures to be instituted for patients enrolled in France.
Amendment 2.2 (France)	08 May 2020	• Updates implemented in this protocol amendment were based on Health Authority feedback.<br>o Removed 2 prohibited medications (ie, rituximab and mitoxantrone) as their use is not contraindicated in the treatment of patients with Coronavirus Disease 2019 severe pneumonia, acute lung injury, or acute respiratory distress syndrome.
Amendment 2.1 (Germany)	08 May 2020	• Updates implemented in this protocol amendment were based on Health Authority feedback. High-level changes were incorporated as follows.<br>o To allow enrollment of patients not currently vaccinated against *Neisseria meningitidis* but who will receive prophylactic antibiotic treatment for at least 8 months after last infusion of study drug or until 2 weeks after they receive vaccination against *N. meningitidis*<br>o Added statement that antivirals may be administered as a part of best supportive care
Amendment 2 (Global)	17 Apr 2020	• Updates implemented in this protocol amendment were based on Health Authority feedback. High-level changes were incorporated as follows.<br>o To relocate “Survival (based on all-cause mortality) at Day 60 and Day 90” from the list of exploratory endpoints to the list of secondary endpoints<br>o To clarify that patients discharged from the hospital before the end of the Primary Evaluation Period will be contacted via telephone on Day 29 to determine health status (eg, survival, mechanical ventilation, hospitalization, intensive care unit, and dialysis)<br>o To remove the definition of Full Analysis Set and add definition of Intent to Treat<br>o To revise analyses of selected endpoints
Amendment 1 (Global)	13 Apr 2020	• To clarify the informed consent process for patients who are unconscious and whose legally acceptable representative is not immediately available<br>• To append Investigator guidance regarding the management of potential drug infusion reactions during ravulizumab administration.
Original Protocol (Global)	09 Apr 2020	Not applicable

10.8. Appendix 8: Abbreviations

Abbreviated or Specialist Term	Explanation
ADA	antidrug antibody
AE	adverse event
aHUS	atypical hemolytic uremic syndrome
ANCOVA	analysis of covariance
ARDS	acute respiratory distress syndrome
BiPAP	bilevel positive airway pressure
BSC	best supportive care
C3	complement component 3
C5	complement component 5
C5a	complement component 5a
C5aR	C5a receptor
C5b-9	terminal complement complex
CIOMS	Council for International Organizations of Medical Sciences
CONSORT	Consolidated Standards of Reporting Trials
CoV	coronavirus
COVID-19	Coronavirus Disease 2019
CPAP	continuous positive airway pressure
CRF	case report form
CT	computed tomography
DMC	Data Monitoring Committee
ECG	electrocardiogram
eCRF	electronic case report form
EDC	electronic data capture
EQ-5D-5L	EuroQol-5 Dimension-5 Level
ET	early terminated/termination
FiO2	fraction of inspired oxygen
FSH	follicle stimulating hormone
GCP	Good Clinical Practice
GCS	Glasgow Coma Scale
GDS	global drug safety
HR	heart rate
HRT	hormonal replacement therapy
hs-TnI	high sensitivity troponin I
HSCT-TMA	thrombotic microangiopathy following hematopoietic stem-cell transplant
IB	Investigator's Brochure
ICF	informed consent form
ICH	International Council for Harmonisation of Technical Requirements for Pharmaceuticals for Human Use
ICU	intensive care unit
ITT	intent-to-treat
IEC	Independent Ethics Committee
IFN	interferon
IL	interleukin
IRB	Institutional Review Board
IV	intravenous
IVIg	intravenous immunoglobulin
KM	Kaplan-Meier
mAb	monoclonal antibody
MAR	missing at random
MASP-2	MBL-associated serine protease-2
MBL	mannose-binding lectin

Abbreviated or Specialist Term	Explanation
MCP	monocyte chemoattractant protein
MCS	Mental Component Summary
MERS-CoV	Middle East respiratory syndrome coronavirus
MH	Mantel-Haenszel
MMRM	mixed model for repeated measures
NT-proBNP	N-terminal pro b-type natriuretic peptide
PaO2	partial pressure of oxygen
PCR	polymerase chain reaction
PCS	Physical Component Summary
PD	pharmacodynamic
PK	pharmacokinetic
PNH	paroxysmal nocturnal hemoglobinuria
PPS	Per Protocol Set
PR	pulse rate
QRS	combination of the Q wave, R wave and S wave
QT	interval between the start of the Q wave and the end of the T wave
QTc	corrected QT interval
RR	respiratory rate
SAE	serious adverse event
SAP	Statistical Analysis Plan
SARS	severe acute respiratory syndrome
SARS-CoV-2	severe acute respiratory syndrome coronavirus 2
SARS-CoV	severe acute respiratory syndrome coronavirus
sC5b-9	soluble C5b-9
SF-12	12-item Short Form
SoA	Schedule of Activities
SOFA	Sequential Organ Failure Assessment
SpO2	peripheral capillary oxygen saturation
SS	Safety Set
SUSAR	suspected unexpected serious adverse reaction
TNF	tumor necrosis factor
TEAE	treatment-emergent adverse event
TESAE	treatment-emergent serious adverse event
VAS	Visual Analogue Scale
WHO	World Health Organization
WOCBP	Woman of childbearing potential

11. REFERENCES

Blackwood B, Ringrow S, Clarke M et al. A core outcome set for critical care ventilation trials. Crit Care Med.2019;47(10):1324-1331.

Casadevall N, Viron B, Kolta A, et al. Pure red-cell aplasia and antierythropoietin antibodies in patients treated with recombinant erythropoietin. N Engl J Med. 2002;346(7):469-475.

Centers for Disease Control and Prevention. Coronavirus Disease 2019 (COVID-19) Situation Summary. https://www.cdc.gov/coronavirus/2019-ncov/summary.html, accessed 10 Mar 2020.

EuroQol Research Foundation. EQ-5D-5L User Guide, 2019. Available from https://euroqol.org/publications/user-guides.

Gralinski LE, Sheahan TP, Morrison TE, et al. Complement activation contributes to severe acute respiratory syndrome coronavirus pathogenesis. mBio. 2018;9(5). doi:10.1128/mBio.01753-18.

Hammerschmidt DE, Weaver LJ, Hudson LD, Craddock PR, Jacob HS. Association of complement activation and elevated plasma-C5a with adult respiratory distress syndrome. Pathophysiological relevance and possible prognostic value. Lancet. 1980;1(8175):947-949.

Hwang IK, Shih WJ, DeCani JS. Group sequential designs using a family of type I error probability spending functions. Stat Med. 1990;9(12):1439-1445.

Ip WKE, Chan KH, Law HKW, et al. Mannose-binding lectin in severe acute respiratory syndrome coronavirus infection. J Infect Dis. 2005;191(10):1697-1704.

Jenkinson C, Layte R, Jenkinson D, et al. A shorter form health survey: can the SF-12 replicate results from the SF-36 longitudinal studies? J Public Health Med. 1997;19(2):179-186.

Jiang Y, Zhao G, Song N, et al. Blockade of the C5a-C5aR axis alleviates lung damage in hDPP4-transgenic mice infected with MERS-CoV. Emerg Microbes Infect. 2018;7:77.

Jodele S, Fukuda T, Mizumo K, Vinks AA, Laskin BL, Goebel J, et al. Variable eculizumab clearance requires pharmacodynamic monitoring to optimize therapy for thrombotic microangiopathy after hematopoietic stem cell transplantation. Biol Blood Marrow Transplant 2016 Feb;22(2);307-315.

Lambden S, Laterre PF, Levy MM et al. The SOFA score - development, utility and challenges of accurate assessment in clinical trials. Crit Care. 2019;23:374.

Lan KKG, DeMets DL. Discrete sequential boundaries for clinical trials. Biometrika. 1983;70(3):659-663.

Li A, Wu Q, Davis C, et al. Transplant-associated thrombotic microangiopathy is a multifactorial disease unresponsive to immunosuppressant withdrawal. Biol Blood Marrow Transplant. 2019;25(3):570-576.

Mantel, N, Haenszel, W. Statistical aspects of analysis of data from retrospective studies of disease. 1959; J Natl Cancer Inst. 1959;22(4):719–748.

Mehta P, McAuley DF, Brown M, et al. COVID-19: consider cytokine storm syndromes and immunosuppression. 2020;395(10229):1033-1034.

Ng WF, To KF, Lam WWL, Ng TK, Lee KC. The comparative pathology of severe acute respiratory syndrome and avian influenza A subtype H5N1-a review. Hum Pathol. 2006;37(4):381-390.

Ohta R, Torii Y, Imai M, Kimura H, Okada N, Ito Y. Serum concentrations of complement anaphylatoxins and proinflammatory mediators in patients with 2009 H1N1 influenza. Microbiol Immunol. 2011;55:191–198.

Pang RTK, Poon TCW, Chan KCA, et al. Serum proteomic fingerprints of adult patients with severe acute respiratory syndrome. Clin Chem. 2006;52(3):421-429.

Pandharipande P, Sanders N, St Jacques P, et al. Calculating SOFA scores when arterial blood gasses are not available: validating SpO2/FiO2 ratios for imputing PaO2/FiO2 ratios in the SOFA score. Crit Care Med. 2006;34(12):A1.

Prodinger W, Wurzner R, Erdei A, Dierich M. Complement. In: Paul WE, editor. Fundamental Immunology. Fourth ed. Philadelphia: Lippincott-Raven; 1999. p. 967-995.

Rota PA. Characterization of a novel coronavirus associated with severe acute respiratory syndrome. Science. 2003;300(5624):1394-1399. doi:10.1126/science.1085952.

Sampson HA, Munoz-Furlong A, Campbell RL, et al. Second symposium on the definition and management of anaphylaxis: summary report--Second National Institute of Allergy and Infectious Disease/Food Allergy and Anaphylaxis Network symposium. J Allergy Clin Immunol. 2006;117(2):391-397.

Sato, T. On the variance estimator of the Mantel-Haenszel risk difference. Letter to the editor. Biometrics; 45:1323–1324. 1989.

Sternbach GL. The Glasgow Coma Scale. J Emerg Med. 2000;19(1):67-71.

Sun S, Zhao G, Liu C, et al. Inhibition of complement activation alleviates acute lung injury induced by highly pathogenic avian influenza H5N1 virus infection. Am J Resp Cell Mol Biol. 2013;49(2):221-230.

Vincent J-L, de Mendonça A, Cantraine F, et al. Use of the SOFA score to assess the incidence of organ dysfunction/failure in intensive care units: Results of a multicenter, prospective study. Crit Care Med. 1998;26(11):1793-1800.

Wang R, Xiao H, Guo R, Li Y, Shen B. The role of C5a in acute lung injury induced by highly pathogenic viral infections. Emerging Microbes Infect. 2015;4(5):e28.

Wang Y, Zhang D, Du G et al. Remdesivir in adults with severe COVID-19: a randomised, double-blind, placebo-controlled, multicentre trial. Lancet. 2020; 20:31022-31029.

Ware JE, Kosinski M, Keller SD. SF-12: How to score the SF-12 physical and mental health summary scales. Boston, MA: The Health Institute, New England Medical Center, Second Edition, 1995.

WHO. Clinical management of severe acute respiratory infection when novel coronavirus (2019-nCoV) infection is suspected. Interim guidance, 28 January 2020.

Wong CK, Lam CWK, Wu AKL, et al. Plasma inflammatory cytokines and chemokines in severe acute respiratory syndrome. Clin Exp Immunol. 2004;136(1):95-103.

Yang YH, Huang YH, Chuang YH, et al. Autoantibodies against human epithelial cells and endothelial cells after severe acute respiratory syndrome (SARS)-associated coronavirus infection. J Med Virol. 2005;77(1):1-7.

Zaki AM, van Boheemen S, Bestebroer TM, Osterhaus ADME, Fouchier RAM. Isolation of a novel coronavirus from a man with pneumonia in Saudi Arabia. N Engl J Med. 2012;367(19):1814-1820.
